# White matter hyperintensities and cholinergic degeneration as Lewy body disease

**DOI:** 10.1002/acn3.52257

**Published:** 2024-12-09

**Authors:** Sungwoo Kang, Seun Jeon, Yeoju Kim, Su‐Hee Jeon, Minsun Choi, Young‐gun Lee, Mijin Yun, Byoung Seok Ye

**Affiliations:** ^1^ Department of Neurology Yonsei University College of Medicine Seoul 03722 Republic of Korea; ^2^ Metabolism‐Dementia Research Institute Yonsei University College of Medicine Seoul 03722 Republic of Korea; ^3^ Department of Neurology, Ilsan Paik Hospital Inje University College of Medicine Goyang 10380 Republic of Korea; ^4^ Department of Nuclear Medicine Yonsei University College of Medicine Seoul 03722 Republic of Korea

## Abstract

**Objective:**

Although basal forebrain (BF) cholinergic degeneration and white matter hyperintensities (WMHs) are important in neurodegeneration in Alzheimer's disease (AD) and dementia with Lewy bodies (DLB), their relationships with dopaminergic degeneration and clinical manifestations remain unclear.

**Methods:**

A total of 407 patients with cognitive impairment meeting the diagnostic criteria for AD, DLB, or both (AD+DLB) were assessed. All participants underwent 3T MRI, dopamine transporter (DAT) positron emission tomography, neuropsychological tests, and assessments for parkinsonism, cognitive fluctuation, visual hallucination, and rapid eye movement sleep behavior disorder (RBD). General linear and logistic regression models were used to investigate the relationships among BF volume, DAT uptake in the anterior caudate (DAT‐AC), WMH volumes in anterior, posterior, periventricular, and deep regions, and clinical manifestations.

**Results:**

DAT‐AC was positively associated with BF volume and negatively associated with anterior periventricular WMH volume, but not with deep WMHs. Both deep and periventricular WMHs volumes were associated with hypertension and the number of microbleeds and lacunae. Lower BF volume and DAT‐AC were independently associated with increased risk of cognitive fluctuation and visual hallucination, whereas lower DAT‐AC was additionally associated with increased risk of RBD and greater parkinsonian severity. Both lower BF volume and DAT‐AC were independently associated with widespread cognitive impairment, whereas higher anterior periventricular WMH volume was associated with executive dysfunction.

**Interpretation:**

BF cholinergic degeneration and anterior periventricular WMHs are closely associated with dopaminergic degeneration. Anterior periventricular WMHs may represent axonal alterations caused by the interplay between Lewy body‐related degeneration and vascular pathologies.

## Introduction

Lewy body disease (LBD), including dementia with Lewy bodies (DLB), is the second most common cause of degenerative dementia, and vascular dementia is the second most common type of dementia after Alzheimer's disease (AD).^1^ LBD is characterized not only by the degeneration of the nigrostriatal dopaminergic system but also by the basal forebrain (BF) cholinergic system,^2^, ^3^, ^4^ which is also vulnerable to AD.^5^ Previous studies have shown that BF atrophy is linked to cognitive dysfunction in both AD and LBD, with an emphasis on memory domains in AD and with broader cognitive domains in LBD.^6^ Moreover, dopaminergic depletion, particularly in the caudate nucleus, is associated with parkinsonism and more severe visuospatial/executive dysfunction in patients with AD,^7^ while it is associated with cognitive dysfunction,^8^, ^9^ cognitive fluctuations, and hallucinations in DLB.^6^ To the best of our knowledge, however, the relationship between dopaminergic depletion, cholinergic degeneration, and clinical symptoms has not been evaluated in cognitively impaired patients, especially considering both AD and LBD.

White matter hyperintensities (WMHs) are core biomarkers of vascular cognitive impairment, particularly subcortical vascular cognitive impairment (SVCI). However, WMHs are frequently observed in patients with AD and/or LBD, and previous studies have shown the role of WMHs in cognitive dysfunction and motor parkinsonism in patients with AD and those with LBD.^10^ Although patients with WMHs are usually considered to have vascular pathology, referred to as vascular parkinsonism or vascular cognitive impairment, recent studies have suggested that WMHs may reflect degenerative pathologies rather than vascular pathology.^11^, ^12^, ^13^, ^14^, ^15^, ^16^ In specific, autopsy studies in AD showed that WMHs could be correspondent to axonal degeneration secondary to neurofibrillary tangles, amyloid pathology such as cerebral amyloid angiopathy (CAA), blood–brain barrier disruption, and neuroinflammation.^17^ In patients with DLB, WMHs are associated with medial temporal lobe atrophy,^18^ cardiovascular autonomic failure,^19^ and vascular risk factors.^16^ However, some studies have shown that more severe periventricular WMHs (PWMHs) are associated with dopamine transporter loss in patients with Parkinson's disease, independent of vascular risk factors,^20^ and associated with the presence of LBD, independent of the presence of AD.^21^ Although patients with LBD have frequent concomitant AD pathology, vascular burden, and BF atrophy, the relationships between WMHs, BF cholinergic atrophy, dopaminergic depletion, and clinical symptoms have yet been evaluated simultaneously considering AD and LBD.

In this study, we evaluated the relationship between striatal dopamine transporter (DAT) uptake, BF volume, WMH burden, and clinical symptoms in patients with carefully phenotyped AD, DLB, and mixed dementia (AD+DLB). We hypothesized that DAT depletion and BF atrophy are closely related and simultaneously contribute to the clinical symptoms of dementia. We also hypothesized that WMH burden could be related to vascular risk factors as well as LBD‐related brain changes, including DAT depletion or BF atrophy.

## Methods

### Study participants

A total of 1490 patients who were referred to the dementia clinic of Yonsei University Severance Hospital, Seoul, Korea, from July 2015 to September 2023 for the evaluation of cognitive decline were recruited. All patients underwent a neurological examination, neuropsychological tests, 3‐T magnetic resonance imaging (MRI), and/or ^18^F‐N‐(3‐fluoropropyl)‐2β‐carboxymethoxy‐3β‐(4‐iodophenyl) nortropane (FP‐CIT) DAT positron emission tomography (PET). Clinical features of LBD, including cognitive fluctuation, visual hallucination, and rapid eye movement behavior disorder (RBD), were assessed using semistructured questionnaires answered by caregivers. Motor parkinsonism was evaluated using the Unified Parkinson's Disease Rating Scale (UPDRS) motor score, which includes subscales for axial symptoms, bradykinesia, rigidity, and tremor.

**Figure 1:**
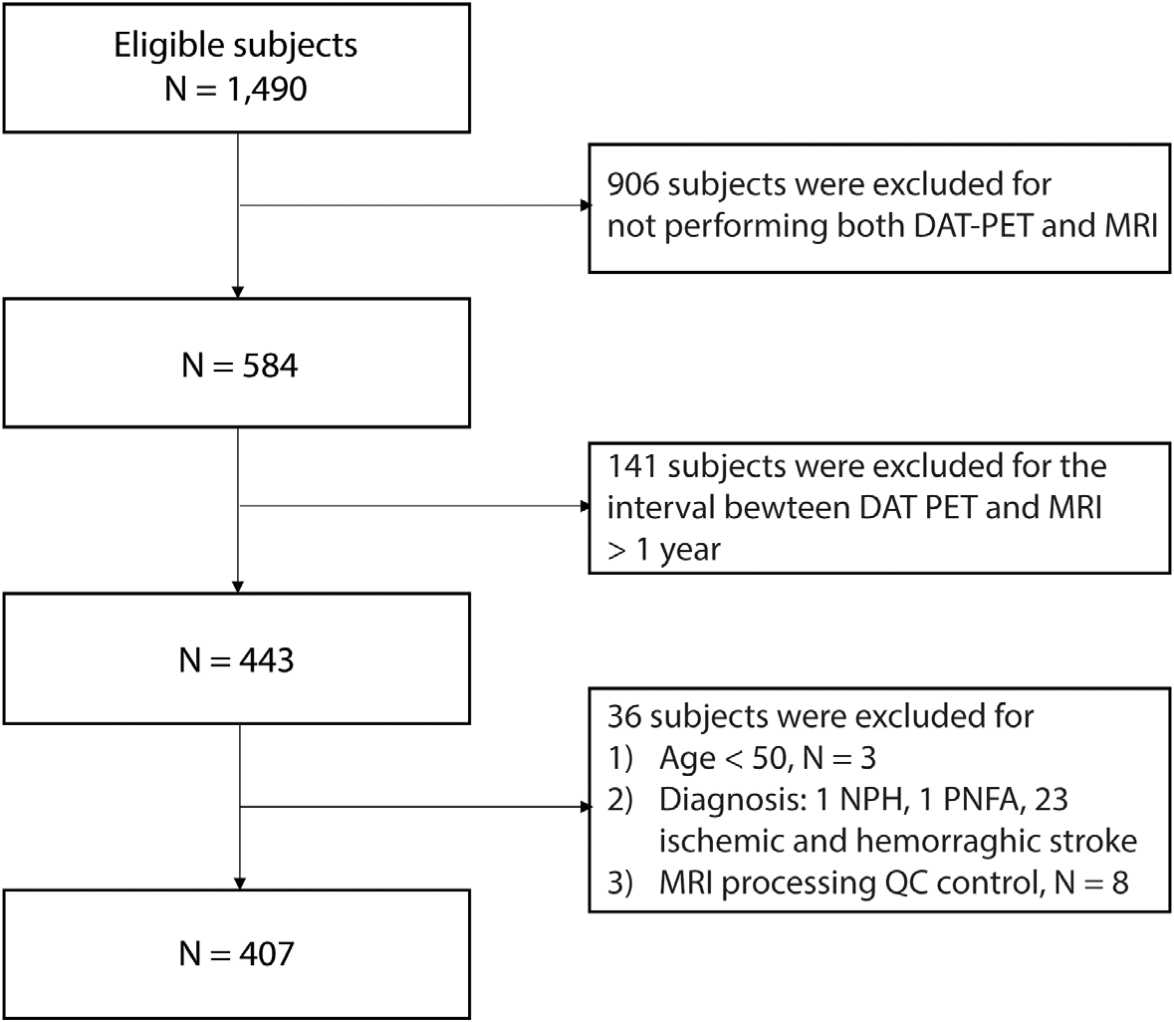
Flow chart of study participants. DAT PET, dopamine transporter positron emission tomography; MRI, magnetic resonance imaging; NPH, normal pressure hydrocephalus; PNFA, progressive nonfluent aphasia; QC, quality control.

We included participants who met the following criteria: (1) underwent both MRI and DAT PET; (2) the interval between the MRI and DAT PET was less than 1 year; and (3) participants were aged 50 years or older. The exclusion criteria were as follows: (1) degenerative causes of dementia other than AD and LBD, including frontotemporal dementia, corticobasal degeneration, and progressive supranuclear palsy; (2) drug‐induced cognitive impairment or parkinsonism; (3) presence of other causes of cognitive impairment, such as epilepsy, psychiatric disorder, normal pressure hydrocephalus, and structural brain lesions (e.g., tumor or hemorrhage); and (4) MRI artifact or image processing error during quality assurance. Finally, 407 patients were included in this study (Fig. 1).

A total of 407 subjects were assessed to determine whether they met the diagnostic criteria for AD or DLB based on clinical symptoms and imaging biomarkers as described previously.^22^ All AD patients met the criteria for probable AD dementia^23^ or AD‐related mild cognitive impairment (MCI)^24^ based on the National Institute on Aging‐Alzheimer's Association workgroup guidelines. All patients with DLB or MCI with Lewy bodies (MCI‐LB) were diagnosed based on the 2017 revised criteria for probable DLB^25^ and the 2020 research criteria for MCI‐LB.^26^ The patients who satisfied the diagnostic criteria of both probable AD and probable DLB were diagnosed with mixed AD/DLB. Therefore, 407 participants were diagnosed as 76 patients with AD, 143 with DLB, and 115 with mixed AD/DLB. The remaining 73 patients had subjective or objective cognitive impairment but did not satisfy the criteria for probable AD or DLB. We did not exclude these patients because we aimed to simultaneously evaluate the effects of AD, DLB, and vascular diseases on brain changes and cognitive dysfunction. There is a possibility that the cognitive impairment in these 73 patients originated from subthreshold AD or DLB, in addition to vascular disease.

Among 407 participants, 121 (29.7%) underwent 18F‐florbetaben (FBB)‐PET scans. In the subgroup of 121 subjects who underwent both FBB and DAT PET scans, 20 were diagnosed with amyloid‐confirmed AD, 29 with amyloid‐confirmed mixed AD/DLB, 6 with amyloid‐positive DLB, and 45 with amyloid‐negative DLB. The remaining 21 participants did not meet the criteria for probable AD or DLB.

Vascular risk factors, including hypertension, diabetes mellitus, and dyslipidemia, were meticulously investigated, and the number of microbleeds and lacunae were counted individually within the entire brain.

The study protocol was approved by the Institutional Review Board of Yonsei University Medical Center (4‐2024‐1288). The need for informed consent was waived due to the retrospective nature of the study.

### Neuropsychological evaluation

All participants completed the standardized Seoul Neuropsychological Screening Battery (SNSB),^27^ which comprises tests of attention, language, visuospatial ability, memory, and frontal/executive functions. Standardized z‐scores were available for all tests, with scores obtained after age‐ and education‐level matching. The following tests were included in our analyses: the Korean version of the Boston Naming Test for the language domain; the copying item of the Rey–Osterrieth Complex Figure Test (RCFT) for the visuospatial domain; the immediate recall, 20‐min delayed recall, and recognition items of the RCFT and Seoul Verbal Learning Test (SVLT) for the memory domain; and the digit span backward, semantic (animal), and phonemic Controlled Oral Word Association Test (COWAT) and Stroop Color Test for the executive domain. The scores in each cognitive test were classified as abnormal when they were >1 standard deviation below the normal values.

### Image acquisition and processing with MRI and PET

The participants were scanned using a Philips 3T MRI scanner (Philips Achieva; Philips Medical Systems, Best, The Netherlands) with a SENSE head coil (SENSE factor = 2). T1‐weighted (T1W) MRI scans were obtained using a three‐dimensional T1W turbo‐field echo sequence with the following parameters: axial acquisition matrix, 224 × 224; reconstructed matrix, 256 × 256 with 170 slices; voxel size, 0.859 × 0.859 × 1 mm3; field of view, 220 mm; echo time, 4.6 ms; repetition time, 9.8 ms; and flip angle, 8°.

FP‐CIT and FBB PET scans were obtained using a Discovery 600 system (General Electric Healthcare, Milwaukee, WI, USA). A dose of 185 MBq (5 mCi) FP‐CIT or 300 MBq (8 mCi) FBB was injected intravenously during the procedure. Ninety minutes after injection, images were acquired over a 20‐min session. The images were reconstructed using the ordered subset expectation–maximization algorithm with four iterations and 32 subsets. A Gaussian filter with a 4‐mm full width at half maximum (FWHM) kernel was applied to the reconstructed PET images, yielding a 256 × 256 matrix with 0.98‐mm pixels and 0.98‐mm slice thickness.

### Quantification of WMH volumes

T1W and FLAIR (fluid‐attenuated inversion recovery) scans were processed using the MINC toolkit (https://bic‐mni.github.io). We corrected the images for intensity nonuniformities and excluded nonbrain tissue followed by co‐registration using a rigid body transformation. T1W images were linearly registered to the Alzheimer's Disease Neuroimaging Initiative (ADNI)‐Montreal Neurological Institute (MNI) atlas, a specific T1W template for the older individuals.^28^ WMH segmentation was performed using a U‐Net convolutional neural network, optimized to highlighting partial‐volume WMHs, thereby enhancing segmentation accuracy.^29^ We classified periventricular WMHs (PWMHs) as those within a 10‐mm sphere along ventricular cerebrospinal fluid edges and deep WMHs (DWMHs) in other white matter areas. These were further divided, based on their anterior/posterior location, using a split point of y = −18 in the MNI space, into four categories: anterior PWMH (PWMH‐A), posterior PWMH (PWMH‐P), anterior DWMH (DWMH‐A), and posterior DWMH (DWMH‐P). For voxel‐based analysis, WMH voxels were nonlinearly transformed into the ADNI‐MNI atlas via each participant's T1W images using the Advanced Normalization Tools Symmetric Normalization (ANTs SyN) registration algorithm and then resampled using nearest‐neighbors before being blurred with an 8‐mm FWHM Gaussian kernel.

### Quantification of BF volume

T1W images were processed using the FMRIB Software Library (FSL, http://www.fmrib.ox.ac.uk/fsl) and the optimized voxel‐based morphometry protocol.^30^ To minimize bias in tissue segmentation due to white matter lesions, these hypo‐intense areas in T1W images were filled with intensities from surrounding normal‐appearing brain tissue on the inhomogeneity‐corrected image as described in a previous study.^31^ The images were then brain‐extracted and segmented into different tissue types (gray matter, white matter, and cerebrospinal fluid), based on a hidden Markov random field model and an associated Expectation–Maximization algorithm, and aligned to the MNI standard space. A study‐specific symmetric template was created, and gray matter images were nonlinearly registered to this template and modulated.^30^ The modulated gray matter images were then smoothed with 4‐mm FWHM Gaussian kernels. The BF region‐of‐interest (ROI) was identified using the Statistical Parametric Mapping Anatomy Toolbox. BF volumes were derived by summing the modulated gray matter voxel values within Ch4 masks, created using a combination of postmortem MRI and histology from 10 autopsied brains.^32^

### Quantification of striatal DAT uptake

ROIs in the striatum were segmented using the FSL FIRST algorithm based on parameterized deformable surface meshes.^33^ FP‐CIT PET scans were co‐registered to individual T1W MRI scans, and standardized uptake value ratio (SUVR) maps of the FP‐CIT PET images were generated using occipital white matter as the reference region. The FP‐CIT PET images were then smoothed using a 6‐mm FWHM Gaussian kernel. Following the anteroposterior axis, we designated the front two‐fifths of the putamen and caudate as the anterior segments and the rear two‐fifths as the posterior segments. The median SUVRs of the FP‐CIT PET scans from these segmented ROIs were calculated including the anterior caudate (DAT‐AC), posterior caudate (DAT‐PC), anterior putamen (DAT‐AP), and posterior putamen (DAT‐PP).

### Quality assurance for image processing

All MRI and PET images and preprocessing outcomes from the automated pipelines were visually inspected for quality assurance by three researchers (SWK, SJ, and BSY) who were blinded to the participants' information. Eight participants were excluded from the initial dataset due to image processing errors and MRI artifacts, resulting in the final inclusion of 407 participants in this study.

### Statistical analysis

Statistical analyses of demographic and clinical data were performed using the R statistical software (version 4.2.1). GLMs were used to evaluate the association among BF volume, WMHs, and striatal DAT uptake after controlling for age, sex, education, vascular factors (hypertension, diabetes mellitus, dyslipidemia, number of microbleeds, and number of lacunae), and intracranial volume. The same analyses for voxel‐wise associations between DAT uptakes and WMHs were performed using the SurfStat toolbox (http://www.math.mcgill.ca/keith/surfstat).

As DAT‐AC had the highest Pearson correlation coefficient with the relationship between BF volume and PWMH‐A (Fig. S1), BF volume, DAT‐AC, and PWMH‐A were used as main predictors in GLMs or logistic models to determine their effects on neuropsychological test z‐scores, parkinsonian motor severity, and risk of cognitive fluctuation, visual hallucination, and RBD after controlling for the same covariates. Explorative analyses of the three‐way or two‐way interactions of BF volume, DAT‐AC, and PWMH‐A on clinical outcomes were conducted by controlling for the same covariates.

The independent effects of AD and DLB on the BF volume, DAT‐AC, and PWMH‐A were investigated using GLMs in the whole participants and in the subgroup who underwent both FBB and DAT PET (Table 1, Table S1) after controlling for the same covariates.

**Table 1:** Demographics of study participants.

Number	407
Age, years	76.9 ± 7.2
Female, *n* (%)	233 (57.2%)
Education	10.3 ± 4.7
Dementia stage, *n* (%)	
SCD	78 (19.2%)
MCI	167 (41.0%)
Dementia	162 (39.8%)
K‐MMSE	23.4 ± 4.1
UPDRS part III motor total score	25.5 ± 14.0
Axial symptoms	5.7 ± 3.9
Bradykinesia	14.1 ± 7.9
Rigidity	4.8 ± 3.4
Tremor	0.4 ± 0.7
DLB clinical features, *n* (%)	
Cognitive fluctuation	197 (48.4%)
Visual hallucination	61 (15.0%)
RBD	134 (32.9%)
Vascular risk factors, *n* (%)	
Hypertension	270 (66.3%)
Diabetes mellitus	123 (30.2%)
Dyslipidemia	224 (55.0%)
Subjects with microbleeds	191 (47.0%)
Subjects with lacunae	116 (28.6%)
Diagnosis, *n* (%)	
Clinical AD	191 (46.9%)
Amyloid‐confirmed AD	49 (12.0%)
DLB	258 (63.4%)
Mixed disease	115 (28.3%)
Subjects who underwent FBB PET, *n* (%)	121 (29.7%)

Data are expressed as means (SD) or numbers (%).

AD, Alzheimer's disease; DLB; dementia with Lewy bodies; FBB PET, ^18^F‐florbetaben positron emission tomography; K‐MMSE, Korean version of the Mini‐Mental State Examination; MCI, mild cognitive impairment; RBD, rapid eye movement sleep behavior disorder; SCD, subjective cognitive decline; UPDRS, Unified Parkinson's Disease Rating Scale.

For above statistical analyses, sensitivity analyses were conducted by excluding 73 patients who did not satisfy the criteria for probable AD or DLB from the whole participants. Also, further analyses were then performed separately for the non‐AD/non‐DLB (*N* = 73), AD (*N* = 76), DLB (*N* = 143), mixed (AD/DLB, *N* = 115), and combined DLB + AD/DLB (*N* = 258) subgroups.

## Results

### Demographic and clinical characteristics of the participants

The demographic and clinical characteristics of the study participants are shown in Table 1. The mean participant age was 76.9 ± 7.2 years, and the mean years of education was 10.3 ± 4.7 years. Of the 407 participants, 270 (66.3%) had hypertension, 123 (30.2%) had diabetes mellitus, and 224 (55.0%) had dyslipidemia. Seventy‐eight participants (19.2%) had subjective cognitive impairment, 167 (41.0%) had mild cognitive impairment, and 162 (39.8%) had dementia. The mean K‐MMSE score was 23.4 ± 4.1. The mean UPDRS motor score at the time of DAT PET was 25.5 ± 14.0. One hundred and ninety‐seven participants (48.4%) had cognitive fluctuation, 61 (15.0%) had visual hallucination, and 134 (32.9%) had RBD. On brain MRI, 191 participants (47%) had microbleeds and 116 (28.6%) had lacunae. One hundred and ninety‐one participants (46.9%) were clinically diagnosed with AD, 258 (63.4%) were diagnosed with DLB, and 115 (28.3%) had mixed AD/DLB. The demographic and clinical characteristics of the subgroup who underwent both FBB and DAT PET (N = 121, 29.7%) are presented in Table S1. The demographic and clinical characteristics of the subgroups (non‐AD/non‐DLB, AD, DLB, and mixed AD/DLB) are shown in Table S2.

### Effect of BF volume and regional WMHs on striatal DAT uptake

**Figure 2:**
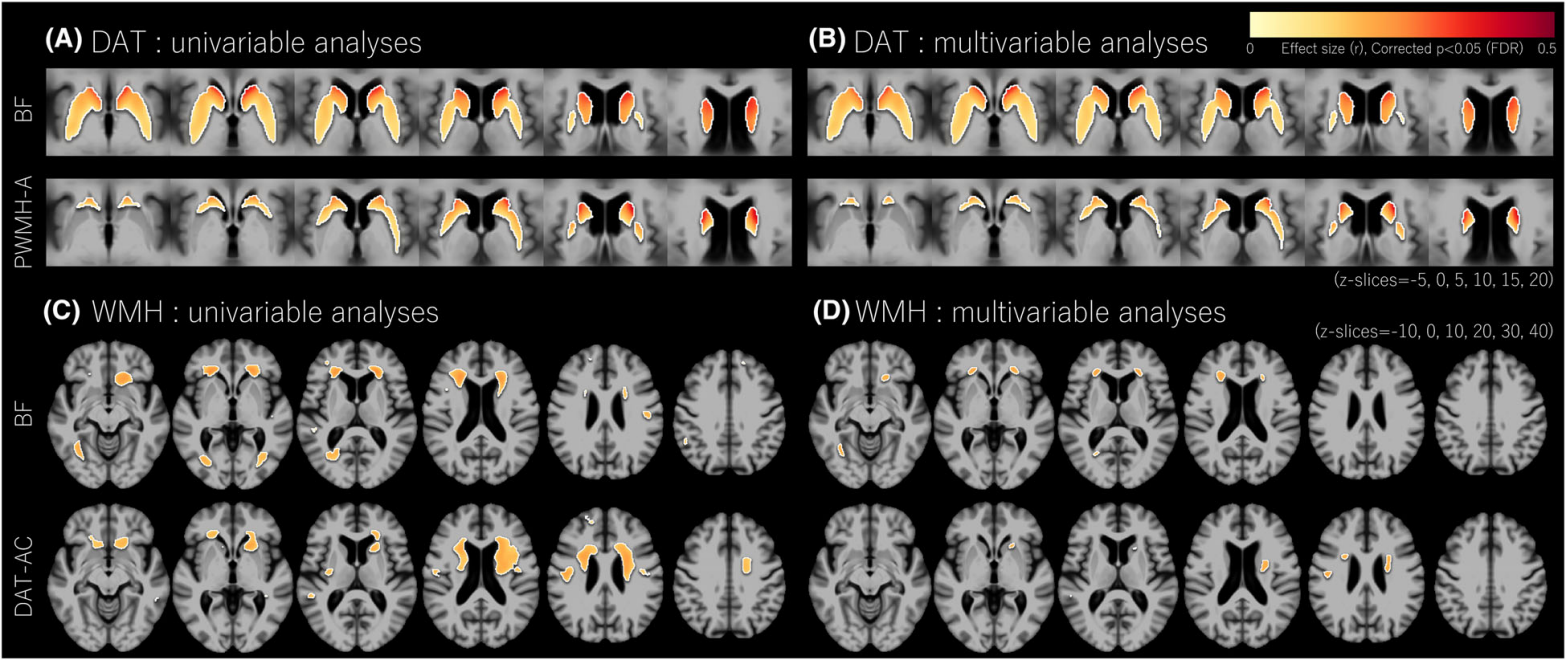
Voxel‐wise analyses for the associations between DAT uptakes and WMHs. Univariable analyses for voxel‐wise DAT uptake using BF volume and PWMH‐A as a predictor (A). Multivariable analysis for voxel‐wise DAT uptake using BF volume and PWMH‐A as predictors (B). Univariable analyses for voxel‐wise WMHs using BF volume and DAT‐AC as a predictor (C). Multivariable analyses for voxel‐wise WMHs using BF volume and DAT‐AC as predictors (D). All analyses were performed after controlling for age, sex, education, intracranial volume, hypertension, diabetes mellitus, dyslipidemia, microbleeds, and lacunes. Negative correlations between WMH and DAT were tested. Effect sizes (r score) were indicated by color intensities within statistically significant regions identified by multiple comparisons correction (false discovery rate [FDR] corrected, *p* < 0.05). AC, anterior caudate; BF, basal forebrain; DAT, dopamine transporter uptake; WMH, white matter hyperintensities; PWMH‐A, anterior periventricular WMH.

Univariable regression analysis showed that decreased BF volume was associated with decreased DAT uptake in all regions (Table S3). Among striatal regions, DAT‐AC had the highest standardized β coefficient for predicting BF volume. Increased PWMH‐A was associated with decreased DAT‐AC. Univariable or multivariable voxel‐wise GLMs showed that decreased BF volume was associated with decreased striatal DAT uptake, with emphasis on the AC, and increased PWMH‐A was associated with decreased DAT uptake in the anterior striatum, with emphasis on the caudate (Fig. 2A,B). The effects of other WMHs on striatal DAT uptake are shown in Figure S2.

### Effect of BF volume, striatal DAT uptake, and vascular factors on regional WMHs

Univariable regression analysis showed that decreased BF volume, decreased caudate DAT, presence of hypertension, increased number of microbleeds, and increased number of lacunae were associated with increased PWMH‐A (Table 2). The BF volume and striatal DAT uptake were not associated with the PWMH‐P or DWMH. The presence of hypertension, increased number of microbleeds, and increased number of lacunae were also associated with increased PWMH‐P and DWMHs.

**Table 2:** Effect of BF volume, striatal DAT uptake, and vascular factors on WMHs.

	PWMH‐A		PWMH‐P		DWMH‐A		DWMH‐P	
	*B*	*p*	*B*	*p*	*B*	*p*	*B*	*p*
Univariable analysis								
BF volume	−0.12	**0.007**	−0.06	0.233	−0.04	0.417	0.03	0.632
DAT								
AP	−0.07	0.109	−0.03	0.558	−0.07	0.175	−0.01	0.907
PP	−0.06	0.151	−0.004	0.939	−0.05	0.322	0.01	0.889
AC	−0.16	**0.001**	−0.08	0.101	−0.11	0.034	−0.05	0.365
PC	−0.12	**0.012**	−0.02	0.670	−0.06	0.215	0.004	0.933
Vascular factors								
Hypertension	0.12	**0.009**	0.12	**0.013**	0.12	**0.017**	0.10	0.047
Diabetes mellitus	0.09	0.050	0.06	0.178	0.005	0.920	0.02	0.709
Dyslipidemia	0.07	0.150	0.05	0.289	0.04	0.448	0.03	0.487
Microbleeds	0.31	**<0.001**	0.33	**<0.001**	0.32	**<0.001**	0.24	**<0.001**
Lacunae	0.39	**<0.001**	0.35	**<0.001**	0.30	**<0.001**	0.30	**<0.001**
Multivariable analysis								
BF volume	−0.09	0.065	−0.04	0.432	−0.01	0.800	0.04	0.450
DAT‐AC	−0.13	**0.006**	−0.07	0.188	−0.10	0.054	−0.06	0.283
Hypertension	0.10	**0.021**	0.10	**0.026**	0.11	**0.026**	0.08	0.106
Diabetes mellitus	0.03	0.439	0.02	0.711	−0.04	0.391	−0.01	0.779
Dyslipidemia	0.03	0.468	0.02	0.669	0.03	0.586	0.02	0.620
Microbleeds	0.27	**<0.001**	0.29	**<0.001**	0.28	**<0.001**	0.20	**<0.001**
Lacunae	0.33	**<0.001**	0.29	**<0.001**	0.24	**<0.001**	0.26	**<0.001**

General linear models were used to investigate the effects of BF volume, striatal DAT, and vascular factors on WMHs, after controlling for age, sex, level of education, and intracranial volume. In the univariable analysis, significant *p*‐values are shown in boldface after false discovery rate correction of 40 regression analyses for multiple comparisons across the 10 predictors and four outcomes. In the multivariable analysis, significant *p*‐values are shown in boldface after false discovery rate correction of the four regression analyses for multiple comparisons across the four outcomes.

B, standardized β coefficient; AC, anterior caudate; AP, anterior putamen; BF, basal forebrain; DAT, dopamine transporter; DWMH‐A, anterior deep white matter hyperintensities; DWMH‐P, posterior DWMH; PC, posterior caudate; PP, posterior putamen; PWMH‐A, anterior periventricular white matter hyperintensities; PWMH‐P, posterior periventricular white matter hyperintensities.

When BF volume, DAT‐AC, and vascular factors were simultaneously entered into GLMs for WMHs, the presence of hypertension, increased number of microbleeds, and increased number of lacunae were associated with increased PWMHs and DWMHs, independent of BF volume and DAT‐AC, except for the presence of hypertension for DWMH‐P. Decreased DAT‐AC was associated with increased PWMH‐A independent of BF volume and vascular factors, whereas decreased BF volume was tended to be associated with PWMH‐A but not with other WMHs.

The univariable and multivariable voxel‐wise GLMs showed that both decreased BF volume and DAT‐AC were associated with higher PWMHs, mainly in the anterior direction (Fig. 2C,D). The effects of other striatal DAT uptakes on WMHs are shown in Figure S2.

### Effect of BF volume, DAT‐AC, and PWMH‐A on LBD features

Univariable regression analysis demonstrated that decreased BF volume was associated with an increased risk of cognitive fluctuation and visual hallucination, and decreased DAT‐AC was further associated with an increased risk of RBD, whereas PWMH‐A was not associated with LBD features (Table 3). Multivariable regression analysis showed that decreased BF volume was independently associated with an increased risk of cognitive fluctuation and visual hallucinations, and decreased DAT‐AC was independently associated with an increased risk of cognitive fluctuation, visual hallucination, and RBD.

**Table 3:** Effect of BF volume, DAT‐AC, and PWMH‐A on DLB features.

	BF volume		DAT‐AC		PWMH‐A	
	OR (95% CI)	*p*	OR (95% CI)	*p*	OR (95% CI)	*p*
Univariable analysis						
Cognitive fluctuation	0.97 (0.96–0.98)	**<0.001**	0.66 (0.55–0.79)	**<0.001**	1.00 (1.00–1.00)	0.129
Visual hallucination	0.97 (0.96–0.98)	**<0.001**	0.55 (0.43–0.70)	**<0.001**	1.00 (1.00–1.00)	0.995
RBD	0.99 (0.98–1.00)	0.151	0.74 (0.62–0.88)	**0.001**	1.00 (1.00–1.00)	0.880
Multivariable analysis						
Cognitive fluctuation	0.97 (0.96–0.98)	**<0.001**	0.74 (0.60–0.89)	**0.002**	1.00 (1.00–1.00)	0.711
Visual hallucination	0.98 (0.96–0.99)	**0.008**	0.60 (0.46–0.78)	**<0.001**	1.00 (1.00–1.00)	0.334
RBD	1.00 (0.99–1.01)	0.559	0.74 (0.61–0.89)	**0.002**	1.00 (1.00–1.00)	0.444

Data are the results of logistic regression models of DLB clinical features (cognitive fluctuation, visual hallucination, and RBD) using BF volume, DAT‐AC, and PWMH‐A as predictors after controlling for age, sex, level of education, hypertension, diabetes mellitus, dyslipidemia, number of microbleeds, number of lacunae, and intracranial volume. Significant *p*‐values are shown in boldface after false discovery rate correction for multiple comparisons of regression analyses for nine univariable analyses and three multivariable analyses.

BF, basal forebrain; CI, confidence interval; DAT‐AC, dopamine transporter uptake in the anterior caudate; DLB, dementia with Lewy bodies; OR, odds ratio; PWMH‐A, anterior periventricular white matter hyperintensities; RBD, rapid eye movement sleep behavior disorder.

Table 4 shows the effects of BF volume, DAT‐AC, and PWMH‐A on UPDRS motor scores, including the subscores of axial symptoms, bradykinesia, rigidity, and tremor. Univariable regression analysis showed that decreased BF volume was associated with higher UPDRS motor scores as well as subscores of bradykinesia; and decreased DAT‐AC was associated with higher UPDRS motor scores and all subscores. Multivariable regression analysis showed that decreased DAT‐AC was associated with UPDRS motor scores and bradykinesia and rigidity subscores, independent of BF volume and PWMH‐A, whereas BF volume and PWMH‐A were not associated with the total UPDRS score or its subscores.

**Table 4:** Effect of BF volume, DAT‐AC, and PWMH‐A on parkinsonism.

	BF volume		DAT‐AC		PWMH‐A	
	*B*	*p*	*B*	*p*	*B*	*p*
Univariable analysis						
Total UPDRS motor score	−0.16	**0.005**	−0.25	**<0.001**	0.09	0.135
Axial	−0.11	0.046	−0.14	**0.015**	0.14	0.025
Bradykinesia	−0.16	**0.005**	−0.25	**<0.001**	0.08	0.214
Rigidity	−0.12	0.042	−0.23	**<0.001**	0.06	0.362
Tremor	−0.09	0.127	−0.12	**0.031**	0.05	0.463
Multivariable analysis						
Total UPDRS motor score	−0.10	0.091	−0.21	**<0.001**	0.04	0.489
Axial	−0.07	0.199	−0.09	0.125	0.11	0.069
Bradykinesia	−0.10	0.087	−0.21	**<0.001**	0.03	0.646
Rigidity	−0.06	0.345	−0.22	**<0.001**	0.01	0.873
Tremor	−0.07	0.266	−0.08	0.208	0.00	0.986

General linear models were used to investigate the effects of BF volume, DAT‐AC, and PWMH‐A on parkinsonism after controlling for age, sex, education, hypertension, diabetes mellitus, dyslipidemia, number of microbleeds, number of lacunae, and intracranial volume. Significant *p*‐values are shown in boldface after false discovery rate correction for multiple comparisons of regression analyses for 15 univariable analyses and 4 multivariable analyses for 4 subscores of the UPDRS motor score.

B, standardized β coefficient; BF, basal forebrain; DAT‐AC, dopamine transporter uptake in the anterior caudate; PWMH‐A, anterior periventricular white matter hyperintensities; UPDRS, Unified Parkinson's Disease Rating Scale.

### Effect of BF volume, DAT‐AC, and PWMH‐A on cognition

Univariable regression analysis demonstrated that decreased BF volume and DAT‐AC and increased PWMH‐A were associated with widespread cognitive dysfunction (Table 5). Multivariable regression analysis showed that the effects of decreased BF volume and DAT‐AC on cognitive dysfunction remained significant except for SVLT recognition and COWAT phonemic in terms of DAT‐AC (Table 6). In contrast, the effects of increased PWMH‐A on cognitive dysfunction were significant only in the digit span backward, COWAT animal, and Stroop color reading tests.

**Table 5:** Univariable analysis of the effect of BF volume, DAT‐AC, and PWMH‐A on cognition.

	BF volume		DAT‐AC		PWMH‐A	
	*B*	*p*	*B*	*p*	*B*	*p*
Digit span backward	0.01	0.882	0.10	0.070	−0.18	**0.004**
K BNT	0.28	**<0.001**	0.23	**<0.001**	−0.18	**0.004**
RCFT copy	0.23	**<0.001**	0.25	**<0.001**	−0.14	**0.023**
SVLT immediate recall	0.32	**<0.001**	0.29	**<0.001**	−0.17	**0.005**
SVLT delayed recall	0.32	**<0.001**	0.23	**<0.001**	−0.19	**0.002**
SVLT recognition	0.25	**<0.001**	0.16	**0.003**	−0.11	0.071
RCFT immediate recall	0.32	**<0.001**	0.24	**<0.001**	−0.12	**0.038**
RCFT delayed recall	0.30	**<0.001**	0.25	**<0.001**	−0.13	**0.030**
RCFT recognition	0.29	**<0.001**	0.22	**<0.001**	−0.16	**0.009**
COWAT animal	0.34	**<0.001**	0.26	**<0.001**	−0.24	**<0.001**
COWAT phonemic	0.18	**0.001**	0.17	**0.002**	−0.13	**0.032**
Stroop color reading	0.32	**<0.001**	0.25	**<0.001**	−0.24	**<0.001**

Univariable general linear models were used to investigate effects of BF volume, DAT‐AC, and PWMH‐A hyperintensities on cognition after controlling for age, sex, education, hypertension, diabetes mellitus, dyslipidemia, number of microbleeds, number of lacunes, and intracranial volume. Significant *p*‐values are shown in boldface after false discovery rate correction for multiple comparisons of the regression analyses of 12 tests. BF, basal forebrain; COWAT, controlled oral word association test; DAT‐AC, dopamine transporter uptake in the anterior caudate; K‐BNT, Korean version of the Boston naming test; PWMH‐A, anterior periventricular white matter hyperintensities; RCFT, Rey–Osterrieth complex figure Test; SVLT, Seoul verbal learning test.

**Table 6:** Multivariable analysis of the effect of BF volume, DAT‐AC, and PWMH‐A on cognition.

	BF volume		DAT‐AC		PWMH‐A	
	*B*	*p*	*B*	*p*	*B*	*p*
Digit span backward	−0.04	0.523	0.09	0.117	−0.17	**0.008**
K‐BNT	0.23	**<0.001**	0.14	**0.016**	−0.12	0.047
RCFT copy	0.16	**0.003**	0.19	**0.001**	−0.08	0.193
SVLT immediate recall	0.25	**<0.001**	0.20	**<0.001**	−0.10	0.101
SVLT delayed recall	0.27	**<0.001**	0.14	**0.014**	−0.13	0.028
SVLT recognition	0.22	**<0.001**	0.09	0.122	−0.06	0.306
RCFT immediate recall	0.27	**<0.001**	0.16	**0.005**	−0.05	0.353
RCFT delayed recall	0.24	**<0.001**	0.17	**0.002**	−0.06	0.302
RCFT recognition	0.24	**<0.001**	0.13	**0.023**	−0.10	0.094
COWAT animal	0.28	**<0.001**	0.15	**0.009**	−0.17	**0.004**
COWAT phonemic	0.14	**0.018**	0.12	0.043	−0.10	0.122
Stroop color reading	0.26	**<0.001**	0.14	**0.011**	−0.17	**0.003**

Multivariable general linear models were used to investigate the effects of BF volume, DAT‐AC, and PWMH‐A on cognition, after controlling for age, sex, education, hypertension, diabetes mellitus, dyslipidemia, number of microbleeds, number of lacunae, and intracranial volume. Significant *p*‐values are shown in boldface after false discovery rate correction for multiple comparisons of the regression analyses of 12 tests.

*B*, standardized β coefficient; BF, basal forebrain; COWAT, Controlled Oral Word Association Test; DAT‐AC, dopamine transporter uptake in the anterior caudate; K‐BNT, Korean version of the Boston Naming Test; PWMH‐A, anterior periventricular white matter hyperintensities; RCFT, Rey–Osterrieth Complex Figure Test; SVLT, Seoul Verbal Learning Test.

Explorative analyses of two‐way or three‐way interactions on all cognitive test scores revealed significant BF volume×DAT‐AC interactions for the K‐BNT and RCFT copy and BF volume×PWMH‐A interactions for the digit span backward (Table S4). Figure S3 demonstrated detrimental synergistic interactions of BF volume×DAT‐AC or BF volume×PWMH‐A on deficits in those tests.

### Independent effect of AD and DLB on BF volume, DAT‐AC, and PWMH‐A

The presence of AD was independently associated with decreased BF volume, whereas the presence of DLB was independently associated with decreased BF volume and DAT‐AC in all participants and the subgroup 1 that underwent both FBB and DAT PET scans (Table S5). When the analysis was limited to the subgroup 1, the presence of DLB was additionally associated with increased PWMH‐A, independent of the presence of AD.

### Sensitivity analyses

Even after excluding 73 patients who did not satisfy the criteria for probable AD or DLB, all the results on sensitivity analyses are very similar to main results (Tables S6–S11).

Figures S4 and S5 and Tables S12–S16 present the results of sensitivity analyses conducted separately for the non‐AD/non‐DLB, AD, DLB, AD/DLB, and combined DLB + AD/DLB subgroups. Overall, the results were consistent with the main findings, with the subgroups containing DLB (either DLB or AD/DLB) appearing to drive the observed effects.

## Discussion

We evaluated the relationships among striatal DAT uptake, BF volume, WMH burden, and clinical symptoms in patients with cognitive impairment due to AD, DLB, or mixed AD/DLB. Our major findings are as follows. First, lower DAT‐AC was associated with lower BF volume independent of vascular factors. Second, lower DAT‐AC was associated with a higher PWMH‐A, but not with DWMHs, independent of the BF volume. Both DWMHs and PWMHs were associated with hypertension and the number of microbleeds and lacunae. Third, both lower BF volume and DAT‐AC were independently associated with an increased risk of cognitive fluctuation and visual hallucination, whereas lower DAT‐AC was additionally associated with an increased risk of RBD and higher UPDRS motor scores. Fourth, lower BF volume and DAT‐AC were independently associated with widespread cognitive dysfunction, whereas higher PWMH‐A was associated with attention/executive dysfunction. Taken together, our results suggest that dopaminergic and cholinergic degeneration are closely related and reflect the underlying LB‐related degeneration. PWMH‐A may be a manifestation of axonal alterations caused by the interplay between Lewy body‐related degeneration and vascular pathology.

Lower striatal DAT uptake, especially in the AC, was associated with a lower BF volume, independent of vascular factors. To the best of our knowledge, no previous studies have evaluated the association between striatal DAT function and cholinergic BF degeneration. Our finding is in line with the previous pathological studies showing that LB pathologies are associated with severe degeneration of the cholinergic neurons in the BF.^2^, ^3^, ^4^ Considering presynaptic α‐synuclein aggregates cause synaptic dysfunction and subsequent loss of dendritic spines in postsynaptic neurons,^34^ destruction of BF cholinergic system might be induced by the spreading of LB‐related degeneration or α‐synuclein from the substantia nigra^35^, ^36^ or caudate nucleus,^37^ whose neurons project directly to BF cholinergic cells. Although both AD and DLB were independently associated with lower BF volume, only DLB was associated with lower DAT‐AC independent of AD (Table S5). Therefore, the association between the DAT‐AC and BF volume was not confounded by the presence of AD. These results emphasize the critical role of nigrostriatal dysfunction in cholinergic degeneration.

Lower DAT‐AC was associated with higher PWMH‐A. In line with our results, previous studies have shown significant association between lower striatal DAT uptake and more severe WMHs in patients with Parkinson's disease (PD)^20^ and clinically normal elderly.^38^ In previous studies, however, although patients with LBD had larger volumes of WMHs than cognitively normal participants,^15^, ^16^ concomitant AD‐related^12^, ^13^, ^14^ or vascular related risk factors were regarded as a major contributor to WMHs rather than LBD pathologies itself.^16^, ^18^, ^39^ However, the association between DAT‐AC and PWMH‐A in our study did not disappear after controlling for vascular risk factors, BF volume, and even the presence of AD (Table S17). As DWMHs were not associated with DAT‐AC, but both DWMHs and PWMHs were correlated with the presence of hypertension and the number of microbleeds and lacunae, our findings could be interpreted as PWMH‐A being an interplay between LBD and SVCI, while DWMH may be primarily related to SVCI.^40^, ^41^ Utilization of DAT imaging, quantitative measurement of DAT uptake and WMH volume, and simultaneous consideration of AD, LBD, and mixed disease could produce different results.

Several mechanisms may explain the association between the DAT‐AC and PWMH‐A. First, glymphatic clearance function, which is affected by degenerative diseases, including AD^42^ and PD,^43^ could be manifested by axonal alterations in the periventricular region. Therefore, the detrimental effects of α‐synuclein or LB‐related pathologies on axonal integrity,^44^ glymphatic dysfunction, or blood–brain barrier disruption^45^, ^46^, ^47^ can be manifested by PWMH‐A. Second, the compromise of the neurovascular unit caused by the degeneration of the BF cholinergic system^48^ could induce microvascular abnormalities reflected by PWMH‐A and confound its association with DAT‐AC. However, the association between the DAT‐AC and PWMH‐A remained significant after controlling for BF volume (Table 2). Moreover, although the association between BF volume and PWMH‐A disappeared after controlling for DAT‐AC, a lower BF volume tended to be associated with a higher PWMH‐A. These results may provide evidence that degenerative processes involving the cholinergic and dopaminergic systems are important for white matter integrity or axonal degeneration reflected by PWMH‐A.^42^, ^43^, ^44^, ^45^, ^46^, ^47^, ^49^

Lower BF volume and DAT‐AC were independently associated with an increased risk of cognitive fluctuation and visual hallucinations, while only lower DAT‐AC was associated with an increased risk of RBD and higher UPDRS total motor scores and bradykinesia and rigidity subscores. These results are consistent with previous studies showing that cholinergic and dopaminergic system have been implicated in the core symptoms of LBD including cognitive fluctuation^50^, ^51^ and visual hallucination.^51^, ^52^ However, our study is the first to evaluate the independent effects of the two predictors on core symptoms of LBD, and the independent contribution of cholinergic degeneration raises the possibility that patients with severe BF atrophy could exhibit visual hallucination or cognitive fluctuation without significant dopaminergic degeneration, which represents a brain‐first rather than body‐first subtype^53^ among multiple distinct subtypes of LBD.^54^ Further studies assessing the cholinergic system and subtypes of LBD are needed to address these issues.

Lower BF volume and DAT‐AC were independently associated with widespread cognitive impairment, and higher PWMH‐A was associated with attention/executive dysfunction. Only a few studies have evaluated the independent effects of cholinergic and dopaminergic degeneration on cognitive dysfunction.^55^, ^56^ In studies performed in patients with PD, dopaminergic and cholinergic degeneration were independently associated with attentional, executive, and memory dysfunction, but not visuospatial dysfunction. In addition, the interaction effects were significant in both attention and executive domains. However, in our study, BF volume and DAT‐AC affected all cognitive domains and had synergistic interaction effects on visuospatial and language dysfunction. The inclusion of patients with AD and mixed disease, along with the use of different methodologies to measure cholinergic and dopaminergic degeneration, may have contributed to the differences in results compared to previous studies. Meanwhile, the effects of DWMHs on cognitive dysfunction were not significant after controlling for DAT‐AC and BF volume (data not shown). Considering that PWMHs could reflect axonal degeneration caused by degenerative disease,^49^ the significant interaction effect of PWMH‐A and BF volume on attention in our study may support the compensatory role of cholinergic neurons in maintaining attention observed in previous studies.^57^, ^58^

We also performed subgroup analyses to explore disease‐specific effects. Sensitivity analyses (Figs. S4 and S5 and Tables S12–S16) showed that lower BF volume was associated with reduced DAT‐AC in the DLB and combined DLB + AD/DLB subgroups. Additionally, a significant negative association between DAT‐AC and anterior PWMHs was observed in the combined DLB + AD/DLB subgroup. Lower DAT‐AC and BF volume were significantly associated with cognitive fluctuations and visual hallucinations in the DLB and AD/DLB subgroups, and lower DAT‐AC was linked to parkinsonism in the DLB and combined DLB + AD/DLB subgroups. In contrast, these associations were less significant in the AD subgroup, possibly due to the small sample size. Since LB‐related neurodegeneration cannot be completely ruled out based solely on clinical symptoms, and given the overlap between AD and DLB pathologies,^59^ analyzing them separately could introduce selection bias. The sensitivity analyses, performed on each subgroup separately, yielded similar patterns, further validating our conclusions. Together, these findings highlight the significant role of dopaminergic degeneration in BF neurodegeneration and its relationship with clinical outcomes, including WMHs.

This study has several limitations. First, among 191 AD diagnoses (76 AD +115 mixed AD/DLB), 49 individuals (25.7%) were confirmed by FBB PET, while the remaining individuals were not. Although the presence of AD was not associated with DAT‐AC or PMWH‐A in the subgroup that underwent both FBB and DAT PET scans (Table S5), future studies are needed to investigate the effect of AD pathologies, including amyloid β or tau burden, on the dopaminergic system and WMHs. Second, we did not exclude 73 subjects who did not satisfy the criteria for probable AD or DLB. It was because we aimed to simultaneously evaluate the effects of AD, LBD, and vascular disease on brain changes and cognitive dysfunction. Moreover, even after excluding these subjects, the effects of AD and DLB on BF volume and DAT‐AC remained significant (Table S5), demonstrating the robustness of the results. Third, this study included participants who visited our dementia clinic with subjective or objective cognitive complaints but not normal controls. Our results, therefore, should be interpreted accordingly. Future studies including normal controls are warranted to investigate the effect of each disease markers on brain changes and clinical manifestations encompassing the transition from healthy aging to neurodegenerative disease.

Our results suggest the importance of dopaminergic and cholinergic degeneration as LB‐related changes in clinical manifestations. Among the WMHs, PWMH‐A may be a manifestation of axonal alterations caused by the interplay between LB‐related degeneration and vascular pathologies.

## Author Contributions

SK, SJ, and BSY contributed to conception and design of the study; SWK, SJ, YK, S‐HJ, MCh, Y‐gL, MY, and BSY contributed to the acquisition and analysis of data; SK, SJ, and BSY contributed to drafting the text or preparing the figures.

## Conflict of Interest

The authors declare that they have no conflict of interest.

## Supporting information


Appendix S1.

## Data Availability

To replicate the procedures and results, any qualified investigator can request anonymized data after obtaining ethics clearance and approval from all authors.
