# Supplementary material for: White matter hyperintensities and cholinergic degeneration as Lewy body disease
Source: Ann Clin Transl Neurol. 2024 Dec 9;12(1):97–109. doi: 10.1002/acn3.52257 (PMC11752093; doi:10.1002/acn3.52257)
Supplement: Supplementary file 1 — Appendix S1. [file ACN3-12-97-s001.docx]

**Supplementary content**

**Supplementary Figure 1.** Pearson correlation analysis between BFV, WMHs, and striatal DAT uptake

**Supplementary Figure 2.** Voxel-wise analyses for the associations between DAT uptakes and WMHs

**Supplementary Figure 3**. Interaction effect of BFV, PWMH-A and DAT-AC on digit span backward, K-BNT, and RCFT copy

**Supplementary Figure 4.** Voxel-wise analyses for the associations of DAT uptake with BF volume and PWMH-A in the AD, DLB, AD/DLB, and combined DLB+AD/DLB subgroups

**Supplementary Figure 5.** Voxel-wise analyses for the associations of WMHs with BF volume and DAT-AC in the combined DLB+AD/DLB subgroup

**Supplementary Table 1**. Demographic of subgroup who performed both FBB and DAT PET

**Supplementary Table 2.** Demographics of the Non-AD/Non-DLB, AD, DLB, and AD/DLB Subgroups

**Supplementary Table 3.** Effect of BFV and WMHs on striatal DAT uptake

**Supplementary Table 4.** Interaction effect of BFV, PWMH-A and DAT-AC on digit span backward, K-BNT, and RCFT copy

**Supplementary Table 5.** Independent effect of AD and DLB on BFV, DAT-AC and PWMH-A

**Supplementary Table 6.** Effect of BFV, striatal DAT uptake, and vascular factors on WMHs after excluding 73 patients who did not satisfy the criteria for probable AD and DLB

**Supplementary Table 7.** Effect of BFV, striatal DAT uptake, and vascular factors on DLB features after excluding 73 patients who did not satisfy the criteria for probable AD and DLB

**Supplementary Table 8.** Effect of BFV, striatal DAT uptake, and vascular factors on parkinsonism after excluding 73 patients who did not satisfy the criteria for probable AD and DLB

**Supplementary Table 9.** Effect of BFV and WMHs on striatal DAT uptake after excluding 73 patients who did not satisfy the criteria for probable AD and DLB

**Supplementary Table 10.** Univariable analysis of the effect of BFV, DAT-AC, and PWMH-A on cognition after excluding 73 patients who did not satisfy the criteria for probable AD and DLB

**Supplementary Table 11.** Multivariable analysis of the effect of BFV, DAT-AC, and PWMH-A on cognition after excluding 73 patients who did not satisfy the criteria for probable AD and DLB

**Supplementary Table 12.** Effect of BF volume and WMHs on striatal DAT uptake in the non-AD/non-DLB, AD, DLB, and AD/DLB subgroups

**Supplementary Table 13**. Effect of BF volume, DAT-AC, and vascular factors on WMHs in the non-AD/non-DLB, AD, DLB, and AD/DLB subgroups and the combined DLB+AD/DLB subgroup

**Supplementary Table 14**. Effect of BF volume, DAT-AC, and anterior PWMHs on DLB features in the non-AD/non-DLB, AD, DLB, and AD/DLB subgroups and the combined DLB+AD/DLB subgroup

**Supplementary Table 15**. Effect of BF volume, DAT-AC, and anterior PWMH on parkinsonism in the non-AD/non-DLB, AD, DLB, and AD/DLB subgroups and the combined DLB+AD/DLB subgroup

**Supplementary Table 16.** Multivariable analysis of the effect of BF volume, DAT-AC, and PWMH-A on cognition in the non-AD/non-DLB, AD, DLB, and AD/DLB subgroups and the combined DLB+AD/DLB subgroup

**Supplementary Table 17.** Independent effect of BFV, striatal DAT uptake, and vascular factors on WMHs after adjusting for presences of AD and DLB

This supplemental material has been provided by the authors to give readers additional information about their work.

**Supplementary Figure 1.** Pearson correlation analysis between BFV, WMHs and striatal DAT uptake


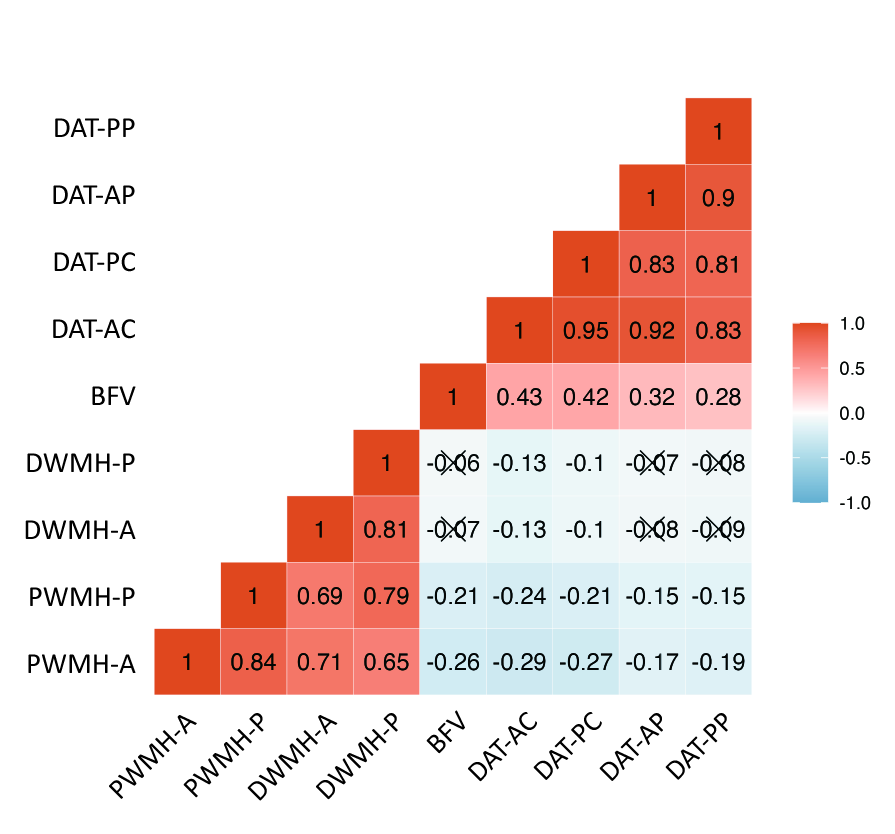


Values representing correlation coefficient and significant correlations (P < 0.05) are displayed without an ‘x’ mark; non-significant values retain an ‘x’ mark. Abbreviations: AC, anterior caudate; AP, anterior putamen; BFV, basal forebrain volume; DAT, dopamine transporter; DWMH-A, anterior deep white matter hyperintensities; DWMH-P, posterior DMWH, PMWH-A, anterior periventricular WMH; PWMH-P, posterior PWMH.

**Supplementary Figure 2.** Voxel-wise analyses for the associations between DAT uptakes and WMHs

**
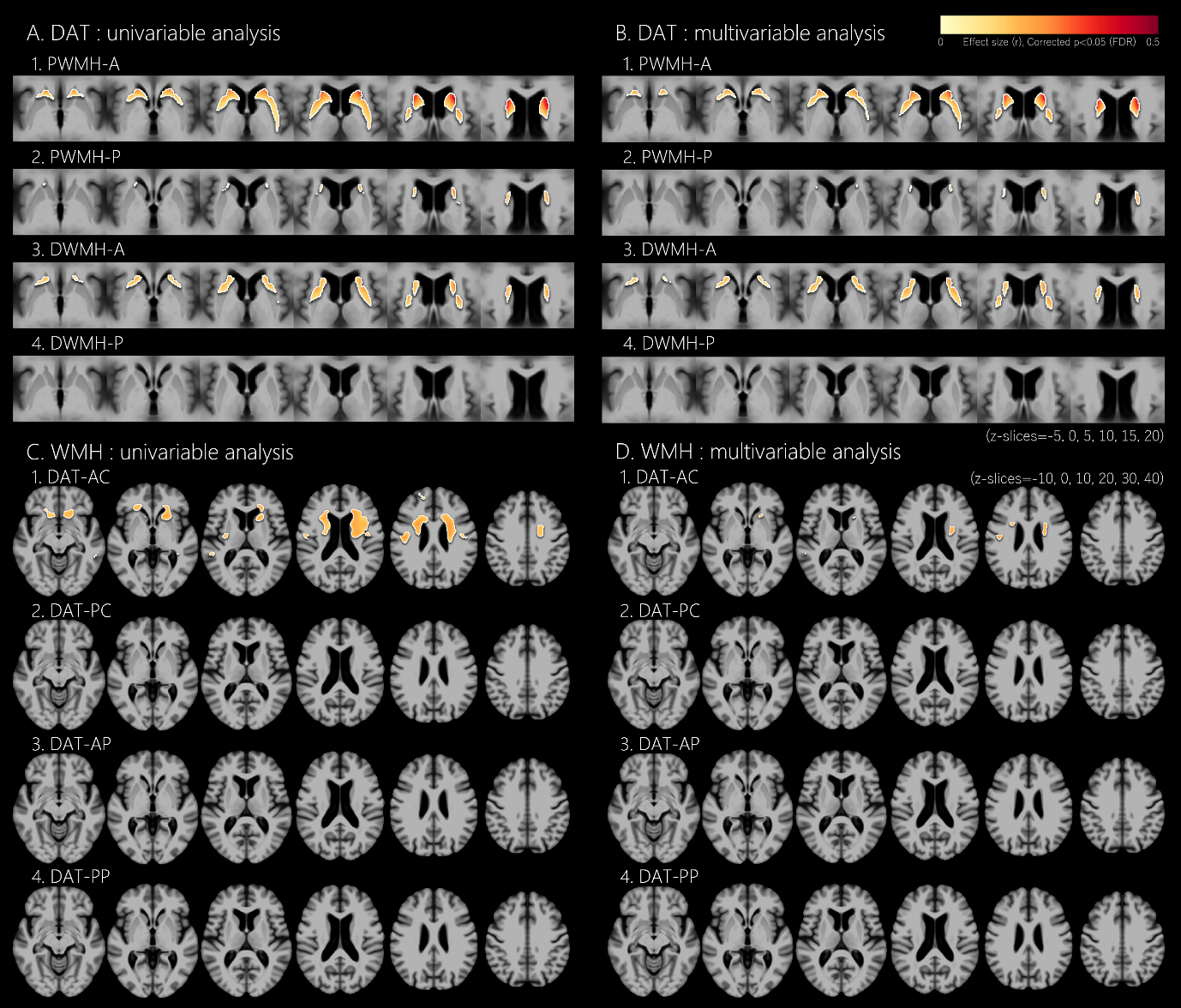
**

Univariable analyses for voxel-wise DAT uptake using one of the four regional WMHs as a predictor (A). Multivariable analysis for DAT uptake using one of the four regional WMHs and BF volume as predictors (B). Univariable analyses for voxel-wise WMHs using one of the four regional DAT uptake as a predictor (C). Multivariable analyses for voxel-wise WMHs using one of the four regional DAT uptake and BF volume as predictors (D). All analyses were performed after controlling for age, sex, education, intracranial volume, hypertension, diabetes, dyslipidemia, microbleeds, and lacunes. Negative correlations between WMH and DAT were tested. Effect sizes (r score) were indicated by color intensities within statistically significant regions identified by multiple comparisons correction (false discovery rate [FDR] corrected, P < 0.05). Abbreviations: AC, anterior caudate; AP, anterior putamen; BF, basal forebrain; DAT, dopamine transporter uptake; WMH, white matter hyperintensities; DWMH-A, anterior deep WMH; DWMH-P, posterior DWMH; PWMH-A, anterior periventricular WMH; PWMH-P, posterior PWMH.

**Supplementary Figure 3**. Interaction effect of BFV, PWMH-A and DAT-AC on digit span backward, K-BNT, and RCFT copy


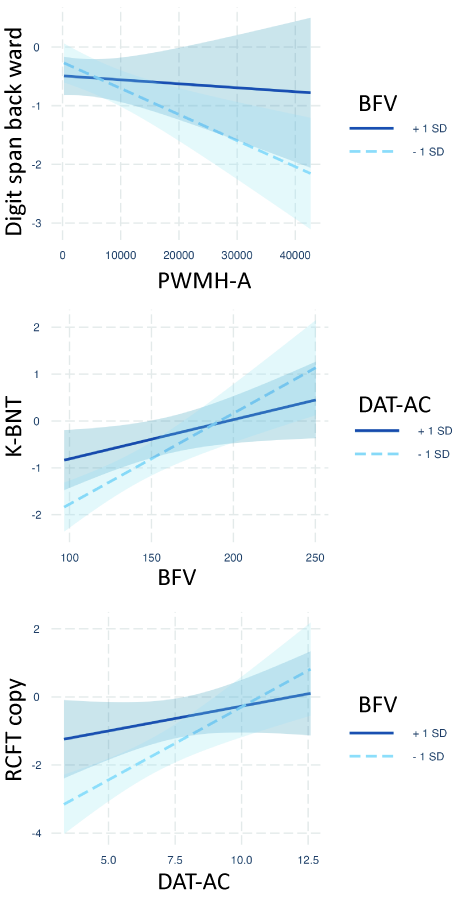


Abbreviations: BFV, basal forebrain volume; DAT-AC, dopamine transporter uptake in the anterior caudate; K-BNT, Korean version of the Boston naming test; PWMH-A, anterior periventricular white matter hyperintensities; RCFT, Rey–Osterrieth complex figure Test.

**Supplementary Figure 4.** Voxel-wise analyses for the associations of DAT uptake with BF volume and PWMH-A in the AD, DLB, AD/DLB, and combined DLB+AD/DLB subgroups

**
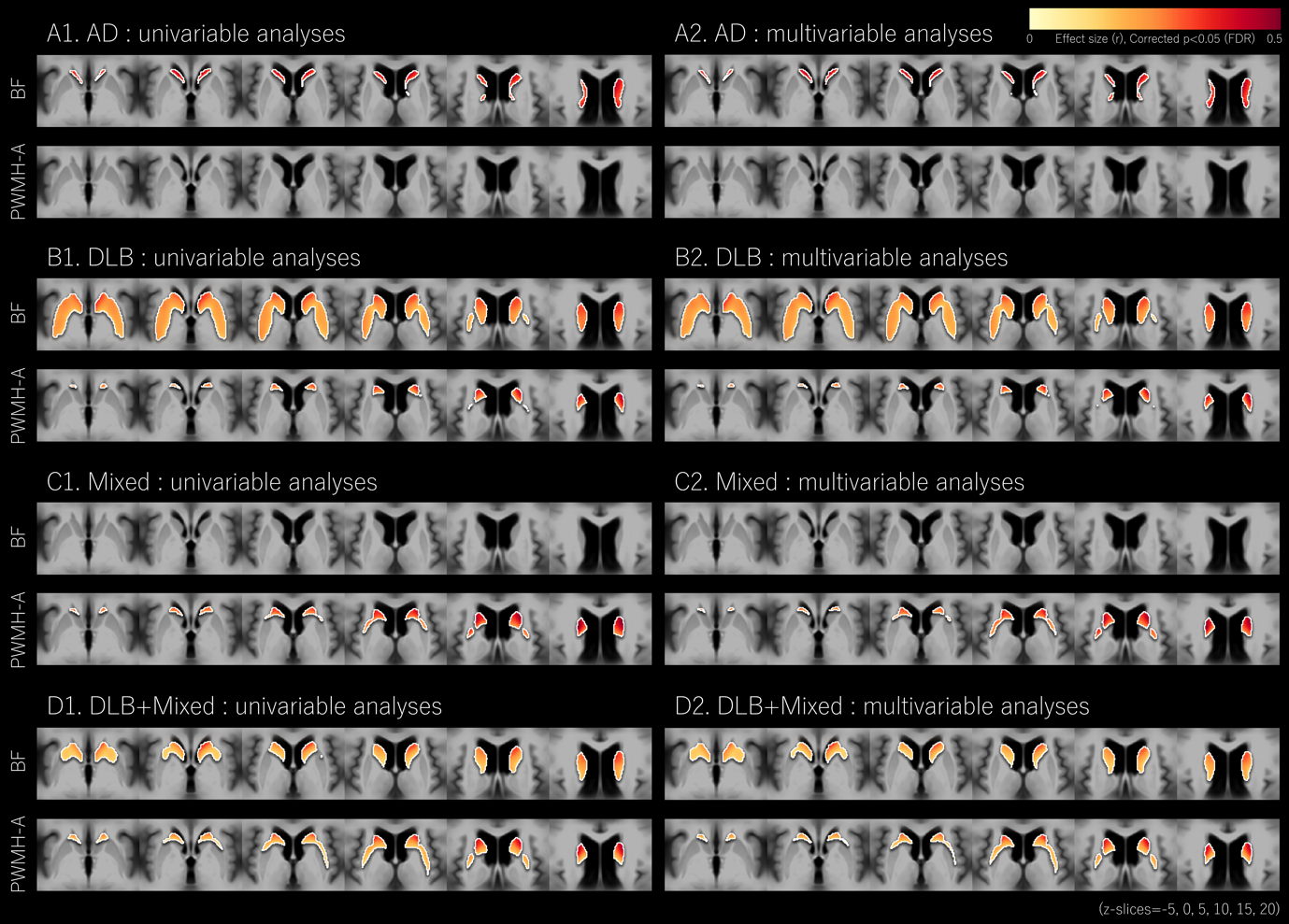
**

Univariable and multivariable analyses for voxel-wise DAT uptake using BF volume and PWMH-A as predictors. There were no significant findings in the non-AD/non-DLB subgroup. All analyses were performed after controlling for age, sex, education, intracranial volume, hypertension, diabetes, dyslipidemia, microbleeds, and lacunes. Negative correlations between WMH and DAT were tested, and positive correlations were tested between BF volume and DAT. Effect sizes (r score) were indicated by color intensities within statistically significant regions identified by multiple comparisons correction (false discovery rate [FDR] corrected, P <0.05). Abbreviations: AC, anterior caudate; BF, basal forebrain; DAT, dopamine transporter uptake; WMHs, white matter hyperintensities; PWMH-A, anterior periventricular WMH.

**Supplementary Figure 5.** Voxel-wise analyses for the associations of WMHs with BF volume and DAT-AC in the combined DLB+AD/DLB subgroup

**
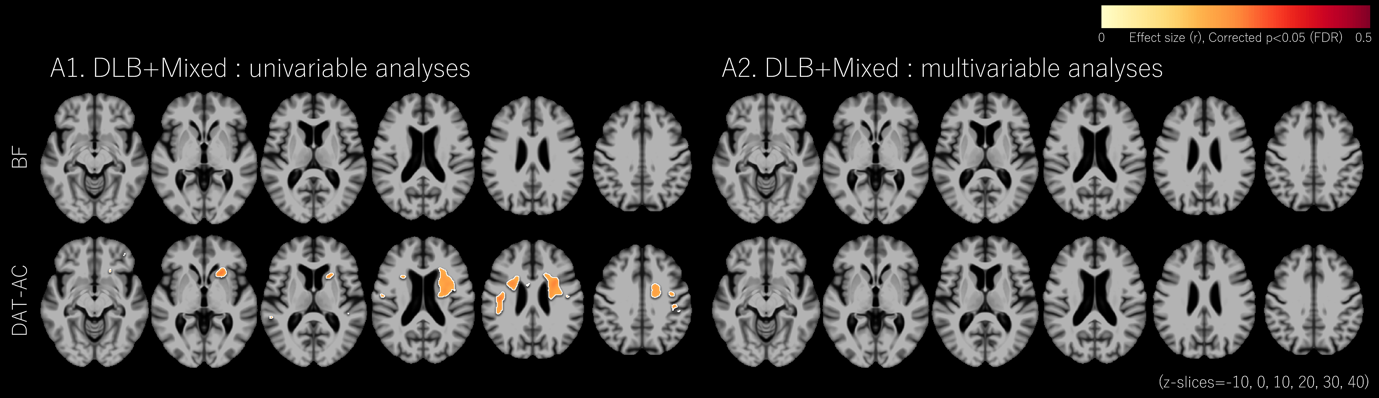
**

Univariable and multivariable analyses for voxel-wise WMHs using BF volume and DAT-AC as predictors. There were significant findings in the non-AD/non-DLB, AD, DLB, and AD/DLB subgroups. All analyses were performed after controlling for age, sex, education, intracranial volume, hypertension, diabetes, dyslipidemia, microbleeds, and lacunes. Negative correlations between WMHs and predictors were tested. Effect sizes (r score) were indicated by color intensities within statistically significant regions identified by multiple comparisons correction (false discovery rate [FDR] corrected, P <0.05). Abbreviations: AC, anterior caudate; BF, basal forebrain; DAT, dopamine transporter uptake; WMHs, white matter hyperintensities.

**Supplementary Table 1**. Demographic of subgroup who performed both FBB and DAT PET

| Number | 121 |
| --- | --- |
| Age | 75.7 ± 7.2 |
| Female, N (%) | 67 (55.4%) |
| Education | 10.0 ± 4.6 |
| **Dementia stage,** N (%) |  |
| SCD | 19 (15.7%) |
| MCI | 45 (37.2%) |
| Dementia | 57 (47.1%) |
| K-MMSE | 22.9 ± 4.0 |
| UPDRS part III motor score | 19.0 ± 13.5 |
| Axial symptoms | 4.0 ± 3.6 |
| Bradykinesia | 10.6 ± 7.9 |
| Rigidity | 3.6 ± 3.3 |
| Tremor | 0.4 ± 0.6 |
| **DLB clinical features**, N (%) |  |
| Cognitive fluctuation | 65 (53.7%) |
| Visual hallucination | 20 (16.5%) |
| RBD | 42 (34.7%) |
| **Vascular risk factors**, N (%) |  |
| Hypertension | 62 (51.2%) |
| Diabetes mellitus | 28 (23.1%) |
| Dyslipidemia | 57 (47.1%) |
| Subjects with microbleeds | 44 (36.7%) |
| Subjects with lacunes | 32 (26.7%) |
| **Diagnosis**, N (%) |  |
| Amyloid confirmed AD | 49 (40.5%) |
| DLB | 80 (66.1%) |
| Mixed disease | 29 (24.0%) |
| **Amyloid positive,** N (%) | 56 (46.3%) |

Data are expressed as means (SD) or numbers (%). Abbreviations: AD, Alzheimer’s disease; DAT, dopamine transporter; DLB, dementia with Lewy bodies; FBB, ^18^F-Florbetaben; K-MMSE, Korean version of mini-mental state examination; MCI, mild cognitive impairment; PET, positron emission tomography; RBD, rapid eye movement sleep behavior disorder; SCD, subjective cognitive decline; UPDRS, unified Parkinson’s disease rating scale.

**Supplementary Table 2.** Demographics of the Non-AD/Non-DLB, AD, DLB, and AD/DLB Subgroups

|  | Non-AD/Non-DLB (N=73) | AD (N=76) | DLB (N=143) | AD/DLB (N=115) | *P* |
| --- | --- | --- | --- | --- | --- |
| Age, y | 74.9 ± 6.7 | 77.0 ± 7.5 | 76.2 ± 7.1 | 78.9 ± 7.1^†※^ | **0.001** |
| Female, n (%) | 51 (69.9%)^※^ | 49 (64.5%)^※^ | 69 (48.3%)^†‡^ | 64 (55.7%) | **0.011** |
| Education | 10.7 ± 4.6 | 10.8 ± 4.7 | 9.7 ± 4.6 | 10.4 ± 4.6 | 0.260 |
| **Dementia stage,** n (%) |  |  |  |  | **< 0.001** |
| SCD | 39 (53.4%) | 14 (18.4%) | 19 (13.3%) | 6 (5.2%) |  |
| MCI | 28 (38.4%) | 40 (52.6%) | 61 (42.7%) | 38 (33%) |  |
| Dementia | 6 (8.2%)^‡※^ | 22 (28.9%)^†^ | 63 (44.1%)^†^ | 71 (61.7%)^†‡※^ |  |
| K-MMSE | 26.2 ± 3.0^‡※^ | 23.9 ± 3.3^†^ | 22.9 ± 4.1^†^ | 21.9 ± 4.4^†‡※^ | **< 0.001** |
| UPDRS part III motor total score | 20.9 ± 13.6 | 23.8 ± 11.0 | 25.9 ± 15.1 | 29.1 ± 13.7^†^ | **< 0.001** |
| Axial symptoms | 4.4 ± 3.6^※^ | 5.3 ± 3.4 | 5.9 ± 3.8^†^ | 6.6 ± 4.3^†^ | **0.001** |
| Bradykinesia | 11.8 ± 7.7 | 13.4 ± 6.7 | 14.2 ± 8.6 | 15.8 ± 7.6^†^ | **0.006** |
| Rigidity | 4.2 ± 3.5 | 4.5 ± 2.5 | 4.9 ± 3.8 | 5.4 ± 3.2 | 0.078 |
| Tremor | 0.4 ± 0.8 | 0.3 ± 0.5 | 0.5 ± 0.6 | 0.5 ± 0.7 | 0.344 |
| **DLB clinical features**, n (%) |  |  |  |  |  |
| Cognitive fluctuation | 0 (0%)^※^ | 0 (0%)^※^ | 101 (70.6%)^†‡^ | 96 (83.5%)^†‡※^ | **< 0.001** |
| Visual hallucination | 0 (0%)^※^ | 0 (0%)^※^ | 39 (27.3%)^†‡^ | 22 (19.1%)^†‡^ | **< 0.001** |
| RBD | 1 (1.4%)^※^ | 3 (3.9%)^※^ | 83 (58%)^†‡^ | 47 (40.9%)^†‡※^ | **< 0.001** |
| **Vascular risk factors**, n (%) |  |  |  |  |  |
| Hypertension | 47 (64.4%) | 47 (61.8%) | 95 (66.4%) | 81 (70.4%) | 0.642 |
| Diabetes mellitus | 23 (31.5%) | 21 (27.6%) | 44 (30.8%) | 35 (30.4%) | 0.956 |
| Dyslipidemia | 46 (63%) | 36 (47.4%) | 86 (60.1%) | 56 (48.7%) | 0.070 |
| Subjects with microbleeds | 33 (45.2%) | 38 (50%) | 63 (44.4%) | 57 (49.6%) | 0.785 |
| Subjects with lacunae | 20 (27.4%) | 18 (23.7%) | 47 (33.1%) | 31 (27%) | 0.473 |

Data are expressed as means ± SD or numbers (%). Group comparisons were performed using the chi-square test or Fisher’s exact test, and analysis of variance as appropriates. ^†^Significantly different from Non-AD/DLB. ^‡^Significantly different from AD. ^※^Significantly different from DLB. Abbreviations: AD, Alzheimer’s disease; DLB; dementia with Lewy bodies; K-MMSE, Korean version of the Mini-Mental State Examination; MCI, mild cognitive impairment; RBD, rapid eye movement sleep behavior disorder; SCD, subjective cognitive decline; UPDRS, Unified Parkinson’s Disease Rating Scale.

**Supplementary Table 3.** Effect of BFV and WMHs on striatal DAT uptake

|  | BFV | | PWMH-A | | PWMH-P | | DWMH-A | | DWMH-P | |
| --- | --- | --- | --- | --- | --- | --- | --- | --- | --- | --- |
|  | B | P | B | P | B | P | B | P | B | P |
| *DAT* |  |  |  |  |  |  |  |  |  |  |
| AP | 0.21 | **< 0.001** | -0.09 | 0.109 | -0.03 | 0.558 | -0.07 | 0.175 | -0.01 | 0.907 |
| PP | 0.16 | **0.003** | -0.08 | 0.151 | -0.004 | 0.939 | -0.05 | 0.322 | 0.01 | 0.889 |
| AC | 0.27 | **< 0.001** | -0.19 | **0.001** | -0.09 | 0.101 | -0.10 | 0.034 | -0.04 | 0.365 |
| PC | 0.25 | **< 0.001** | -0.14 | 0.012 | -0.02 | 0.670 | -0.06 | 0.215 | 0.004 | 0.933 |

Univariable general linear models were used to investigate effects of BFV and WMHs on striatal DAT uptake after controlling for age, sex, education, HTN, DM, dyslipidemia, number of microbleeds, number of lacunes, and intracranial volume. Significant P-values are shown in boldface after false discovery rate correction for multiple comparisons of regressions analyses for 20 tests. Abbreviations: AC, anterior caudate; AP, anterior putamen; BFV, basal forebrain volume; DAT, dopamine transporter uptake; DWMH-A, anterior deep white matter hyperintensities; DWMH-P, posterior DWMH; PC, posterior caudate; PP, posterior putamen; PWMH-A, anterior periventricular WMH; PWMH-P, posterior PWMH.

**Supplementary Table 4.** Interaction effect of BFV, PWMH-A and DAT-AC on digit span backward, K-BNT, and RCFT copy

|  | BFV | | DAT-AC | | PWMH-A | | BFV*DAT-AC | | BFV*PWMH-A | | DAT-AC*PWMH_A | |
| --- | --- | --- | --- | --- | --- | --- | --- | --- | --- | --- | --- | --- |
|  | B | P | B | P | B | P | B | P | B | P | B | P |
| Digit span Backward^*^ | -0.14 | 0.070 | 0.11 | 0.077 | -0.90 | **0.011** |  | N.S | 0.73 | **0.034** |  | N.S. |
| K-BNT^*^ | 0.73 | **0.004** | 0.73 | **0.014** | -0.12 | 0.045 | -0.94 | **0.042** |  | N.S. |  | N.S. |
| RCFT copy^*^ | 0.65 | **0.008** | 0.75 | **0.008** | -0.08 | 0.187 | -0.90 | **0.043** |  | N.S. |  | N.S. |

Multivariable general linear models were used to investigate effects of BFV, DAT-AC and PWMH-A on cognition after controlling for age, sex, education, HTN, DM, dyslipidemia, number of microbleeds, number of lacunes, and intracranial volume. ^*^As the interaction terms of BFV*PWMH for digit span backward and BFV*DAT-AC for K-BNT and RCFT copy tests were significant, interaction terms were simultaneously entered as predictors. Significant P-values are shown in boldface after false discovery rate correction for multiple comparisons of regressions analyses for K-BNT and RCFT copy tests. Abbreviations: BFV, basal forebrain volume; DAT-AC, dopamine transporter uptake in the anterior caudate; K-BNT, Korean version of the Boston naming test; PWMH-A, anterior periventricular white matter hyperintensities; RCFT, Rey–Osterrieth complex figure Test.

**Supplementary Table 5.** Independent effect of AD and DLB on BFV, DAT-AC and PWMH-A

|  | AD | | | | DLB | | | |
| --- | --- | --- | --- | --- | --- | --- | --- | --- |
|  | Beta | SE | B | P | Beta | SE | B | P |
| **Whole subjects, N=407** | | |  |  |  |  |  |  |
| BFV | -7.69 | 2.11 | -0.16 | **< 0.001** | -10.68 | 2.18 | -0.21 | **< 0.001** |
| DAT-AC | 0.11 | 0.12 | 0.04 | 0.387 | -0.59 | 0.13 | -0.20 | **< 0.001** |
| PWMH-A | 116.69 | 556.58 | 0.01 | 0.834 | 744.40 | 573.28 | 0.05 | 0.195 |
| **Subgroup 1, N=121** | | | |  |  |  |  |  |
| BFV | -7.81 | 3.61 | -0.18 | **0.033** | -10.45 | 3.78 | -0.23 | **0.007** |
| DAT-AC | 0.08 | 0.24 | 0.03 | 0.736 | -0.78 | 0.25 | -0.26 | **0.002** |
| PWMH-A | -44.39 | 853.48 | -0.003 | 0.959 | 2018.99 | 893.60 | 0.15 | **0.026** |
| **Subgroup 2, N=334** | | |  |  |  |  |  |  |
| BFV | -6.85 | 2.64 | -0.15 | **0.010** | -10.09 | 3.05 | -0.18 | **0.001** |
| DAT-AC | 0.02 | 0.16 | 0.01 | 0.895 | -0.73 | 0.18 | -0.22 | **< 0.001** |
| PWMH-A | -41.81 | 713.40 | -0.003 | 0.953 | 749.30 | 823.20 | 0.05 | 0.363 |
| **Subgroup 3, N=100** | |  |  |  |  |  |  |  |
| BFV | -11.59 | 4.70 | -0.26 | **0.016** | -15.47 | 5.83 | -0.28 | **0.009** |
| DAT-AC | -0.11 | 0.31 | -0.04 | 0.723 | -1.09 | 0.38 | -0.31 | **0.006** |
| PWMH-A | -454.50 | 1132.00 | -0.04 | 0.689 | 1561.00 | 1406.00 | 0.10 | 0.270 |

Multivariable general linear models were used to investigate effects of presence of AD and DLB on BFV, DAT-AC, and PWMH-A after controlling for age, sex, education, HTN, DM, dyslipidemia, number of microbleeds, number of lacunes, and intracranial volume. The subjects in subgroup 1 additionally performed FBB-PET. Subgroup 2 consisted of subjects excluding 73 subjects who did not meet the criteria of probable AD or DLB from whole participants. Subgroup 3 consisted of subjects excluding 21 subjects who did not meet the criteria of probable AD or DLB from subgroup 1. Significant P-values are shown in boldface after false discovery rate correction for multiple comparisons of 3 regression analyses for BFV, DAT-AC and PWMH-A. Abbreviations: AD, Alzheimer’s disease; B, standardized beta coefficient; BFV, basal forebrain volume; DAT-AC; dopamine transporter uptake in the anterior caudate; DLB, dementia with Lewy bodies; PWMH-A; anterior periventricular white matter hyperintensities; SE, standard error

**Supplementary Table 6.** Effect of BFV, striatal DAT uptake, and vascular factors on WMHs after excluding 73 patients who did not satisfy the criteria for probable AD and DLB

|  | PWMH-A | | PWMH-P | | DWMH-A | | DWMH-P | |
| --- | --- | --- | --- | --- | --- | --- | --- | --- |
| Predictors | B | *P* | B | *P* | B | *P* | B | *P* |
| **Univariable analysis** |  |  |  |  |  |  |  |  |
| BFV | -0.11 | 0.037 | -0.06 | 0.258 | -0.01 | 0.818 | 0.03 | 0.596 |
| *DAT* |  |  |  |  |  |  |  |  |
| AP | -0.09 | 0.105 | -0.05 | 0.399 | -0.07 | 0.225 | -0.03 | 0.551 |
| PP | -0.09 | 0.095 | -0.03 | 0.575 | -0.07 | 0.231 | -0.03 | 0.640 |
| AC | -0.20 | **0.001** | -0.13 | 0.028 | -0.13 | 0.028 | -0.10 | 0.101 |
| PC | -0.17 | **0.004** | -0.08 | 0.156 | -0.10 | 0.103 | -0.05 | 0.388 |
| *Vascular factors* |  |  |  |  |  |  |  |  |
| Hypertension | 0.09 | 0.076 | 0.10 | 0.065 | 0.11 | 0.059 | 0.09 | 0.098 |
| Diabetes mellitus | 0.07 | 0.181 | 0.03 | 0.575 | -0.01 | 0.868 | -0.01 | 0.854 |
| Dyslipidemia | 0.03 | 0.610 | 0.02 | 0.770 | 0.03 | 0.603 | 0.02 | 0.730 |
| Microbleeds | 0.30 | **< 0.001** | 0.35 | **< 0.001** | 0.36 | **< 0.001** | 0.29 | **< 0.001** |
| Lacunae | 0.36 | **< 0.001** | 0.29 | **< 0.001** | 0.25 | **< 0.001** | 0.24 | **< 0.001** |
| **Multivariable analysis** |  |  |  |  |  |  |  |  |
| BFV | -0.07 | 0.206 | -0.03 | 0.535 | 0.02 | 0.710 | 0.06 | 0.348 |
| DAT-AC | -0.15 | **0.006** | -0.09 | 0.091 | -0.13 | 0.033 | -0.09 | 0.122 |
| Hypertension | 0.10 | **0.033** | 0.12 | **0.016** | 0.13 | **0.018** | 0.10 | 0.059 |
| Diabetes mellitus | 0.03 | 0.523 | -0.003 | 0.955 | -0.04 | 0.399 | -0.04 | 0.516 |
| Dyslipidemia | 0.01 | 0.774 | 0.01 | 0.904 | 0.04 | 0.503 | 0.02 | 0.663 |
| Microbleeds | 0.27 | **< 0.001** | 0.33 | **< 0.001** | 0.35 | **< 0.001** | 0.27 | **< 0.001** |
| Lacunae | 0.30 | **< 0.001** | 0.23 | **< 0.001** | 0.19 | **< 0.001** | 0.19 | **< 0.001** |

General linear models were used to investigate the effects of BFV, striatal DAT, and vascular factors on WMHs, after controlling for age, sex, level of education, and intracranial volume. In the univariable analysis, significant *P*-values are shown in boldface after false discovery rate correction of 40 regression analyses for multiple comparisons across the 10 predictors and four outcomes. In the multivariable analysis, significant *P*-values are shown in boldface after false discovery rate correction of the four regression analyses for multiple comparisons across the four outcomes. Abbreviations: B, standardized β coefficient; AC, anterior caudate; AP, anterior putamen; BFV, basal forebrain volume; DAT, dopamine transporter; DWMH-A, anterior deep white matter hyperintensities; DWMH-P, posterior DWMH; PC, posterior caudate; PP, posterior putamen; PWMH-A, anterior periventricular white matter hyperintensities; PWMH-P, posterior periventricular white matter hyperintensities.

**Supplementary Table 7.** Effect of BFV, striatal DAT uptake, and vascular factors on DLB features after excluding 73 patients who did not satisfy the criteria for probable AD and DLB

|  | BFV | | DAT-AC | | PWMH-A | |
| --- | --- | --- | --- | --- | --- | --- |
|  | OR (95% CI) | *P* | OR (95% CI) | *P* | OR (95% CI) | *P* |
| **Univariable analysis** |  |  |  |  |  |  |
| Cognitive fluctuation | 0.97 (0.96~0.99) | **<0.001** | 0.66 (0.54~0.80) | **<0.001** | 1.00 (1.00~1.00) | 0.170 |
| Visual hallucination | 0.98 (0.96~0.99) | **0.002** | 0.58 (0.45~0.74) | **<0.001** | 1.00 (1.00~1.00) | 0.878 |
| RBD | 1.00 (0.99~1.01) | 0.657 | 0.76 (0.63~0.91) | **0.004** | 1.00 (1.00~1.00) | 0.630 |
| **Multivariable analysis** |  |  |  |  |  |  |
| Cognitive fluctuation | 0.98 (0.97~0.99) | **0.001** | 0.72 (0.58~0.89) | **0.002** | 1.00 (1.00~1.00) | 0.705 |
| Visual hallucination | 0.98 (0.97~1.00) | 0.044 | 0.61 (0.47~0.79) | **<0.001** | 1.00 (1.00~1.00) | 0.282 |
| RBD | 1.00 (0.99~1.01) | 0.732 | 0.74 (0.60~0.90) | **0.003** | 1.00 (1.00~1.00) | 0.313 |

Data are the results of logistic regression models of DLB clinical features (cognitive fluctuation, visual hallucination, and RBD) using BFV, DAT-AC, and PWMH-A as predictors after controlling for age, sex, level of education, HTN, DM, dyslipidemia, number of microbleeds, number of lacunae, and intracranial volume. Significant P-values are shown in boldface after false discovery rate correction for multiple comparisons of regression analyses for 9 univariable analyses and 3 multivariable analyses. Abbreviations: BFV, basal forebrain volume; CI, confidence interval; DAT-AC, dopamine transporter uptake in the anterior caudate; DLB, dementia with Lewy bodies; OR, odds ratio; PWMH-A, anterior periventricular white matter hyperintensities; RBD, rapid eye movement sleep behavior disorder.

**Supplementary Table 8.** Effect of BFV, striatal DAT uptake, and vascular factors on parkinsonism after excluding 73 patients who did not satisfy the criteria for probable AD and DLB

|  | BFV | | DAT-AC | | PWMH-A | |
| --- | --- | --- | --- | --- | --- | --- |
|  | B | *P* | B | *P* | B | *P* |
| **Univariable analysis** |  |  |  |  |  |  |
| Total UPDRS motor score | -0.17 | **0.008** | -0.24 | **< 0.001** | 0.11 | 0.113 |
| Axial | -0.11 | 0.072 | -0.13 | 0.036 | 0.13 | 0.044 |
| Bradykinesia | -0.19 | **0.003** | -0.22 | **< 0.001** | 0.10 | 0.132 |
| Rigidity | -0.09 | 0.170 | -0.24 | **< 0.001** | 0.09 | 0.192 |
| Tremor | -0.08 | 0.199 | -0.08 | 0.206 | 0.03 | 0.658 |
| **Multivariable analysis** |  |  |  |  |  |  |
| Total UPDRS motor score | -0.11 | 0.101 | -0.20 | **0.002** | 0.06 | 0.386 |
| Axial | -0.08 | 0.238 | -0.09 | 0.162 | 0.11 | 0.105 |
| Bradykinesia | -0.13 | 0.044 | -0.18 | **0.006** | 0.06 | 0.414 |
| Rigidity | -0.02 | 0.782 | -0.23 | **< 0.001** | 0.04 | 0.519 |
| Tremor | -0.06 | 0.339 | -0.06 | 0.367 | 0.01 | 0.861 |

General linear models were used to investigate the effects of BFV, DAT-AC, and PWMH-A on parkinsonism after controlling for age, sex, education, hypertension, diabetes mellitus, dyslipidemia, number of microbleeds, number of lacunae, and intracranial volume. Significant *P*-values are shown in boldface after false discovery rate correction for multiple comparisons of regression analyses for 15 univariable analyses and 4 multivariable analyses for 4 subscores of the UPDRS motor score. Abbreviations: B, standardized β coefficient; BFV, basal forebrain volume; DAT-AC, dopamine transporter uptake in the anterior caudate; PWMH-A, anterior periventricular white matter hyperintensities; UPDRS, Unified Parkinson’s Disease Rating Scale.

**Supplementary Table 9.** Effect of BFV and WMHs on striatal DAT uptake after excluding 73 patients who did not satisfy the criteria for probable AD and DLB

|  | BFV | | PWMH-A | | PWMH-P | | DWMH-A | | DWMH-P | |
| --- | --- | --- | --- | --- | --- | --- | --- | --- | --- | --- |
|  | B | P | B | P | B | P | B | P | B | P |
| *DAT* |  |  |  |  |  |  |  |  |  |  |
| AP | 0.19 | **< 0.001** | -0.09 | 0.082 | -0.05 | 0.330 | -0.08 | 0.127 | -0.03 | 0.548 |
| PP | 0.13 | 0.014 | -0.06 | 0.183 | -0.01 | 0.819 | -0.06 | 0.263 | -0.01 | 0.881 |
| AC | 0.28 | **< 0.001** | -0.16 | **0.001** | -0.10 | 0.052 | -0.12 | 0.034 | -0.08 | 0.181 |
| PC | 0.26 | **< 0.001** | -0.12 | 0.023 | -0.04 | 0.469 | -0.07 | 0.246 | -0.02 | 0.796 |

Univariable general linear models were used to investigate effects of BFV and WMHs on striatal DAT uptake after controlling for age, sex, education, HTN, DM, dyslipidemia, number of microbleeds, number of lacunes, and intracranial volume. Significant P-values are shown in boldface after false discovery rate correction for multiple comparisons of regressions analyses for 20 tests. Abbreviations: AC, anterior caudate; AP, anterior putamen; BFV, basal forebrain volume; DAT, dopamine transporter uptake; DWMH-A, anterior deep white matter hyperintensities; DWMH-P, posterior DWMH; PC, posterior caudate; PP, posterior putamen; PWMH-A, anterior periventricular WMH; PWMH-P, posterior PWMH

**Supplementary Table 10.** Univariable analysis of the effect of BFV, DAT-AC, and PWMH-A on cognition after excluding 73 patients who did not satisfy the criteria for probable AD and DLB

|  | BFV | | DAT-AC | | PWMH-A | |
| --- | --- | --- | --- | --- | --- | --- |
|  | B | P | B | P | B | P |
| Digit span Backward | -0.02 | 0.723 | 0.10 | 0.108 | -0.19 | **0.005** |
| K BNT | 0.24 | **< 0.001** | 0.19 | **0.002** | -0.18 | **0.007** |
| RCFT copy | 0.18 | **0.003** | 0.24 | **< 0.001** | -0.11 | 0.076 |
| SVLT immediate recall | 0.28 | **< 0.001** | 0.25 | **< 0.001** | -0.14 | **0.028** |
| SVLT delayed recall | 0.26 | **< 0.001** | 0.17 | **0.006** | -0.18 | **0.006** |
| SVLT recognition | 0.19 | **0.002** | 0.12 | 0.053 | -0.10 | 0.120 |
| RCFT immediate recall | 0.28 | **< 0.001** | 0.19 | **0.001** | -0.14 | **0.030** |
| RCFT delayed recall | 0.26 | **< 0.001** | 0.18 | **0.001** | -0.12 | 0.047 |
| RCFT recognition | 0.26 | **< 0.001** | 0.19 | **0.002** | -0.15 | **0.023** |
| COWAT animal | 0.33 | **< 0.001** | 0.21 | **< 0.001** | -0.26 | **< 0.001** |
| COWAT phonemic | 0.16 | **0.011** | 0.15 | **0.014** | -0.12 | 0.074 |
| Stroop color reading | 0.30 | **< 0.001** | 0.27 | **< 0.001** | -0.23 | **0.001** |

Univariable general linear models were used to investigate effects of BFV, DAT-AC, and PWMH-A on cognition after controlling for age, sex, education, HTN, DM, dyslipidemia, number of microbleeds, number of lacunes, and intracranial volume. Abbreviations: BFV, basal forebrain volume; COWAT, controlled oral word association test; DAT-AC, dopamine transporter uptake in the anterior caudate; K-BNT, Korean version of the Boston naming test; PWMH-A, anterior periventricular white matter hyperintensities; RCFT, Rey–Osterrieth complex figure Test; SVLT, Seoul verbal learning test

**Supplementary Table 11.** Multivariable analysis of the effect of BFV, DAT-AC, and PWMH-A on cognition after excluding 73 patients who did not satisfy the criteria for probable AD and DLB

|  | BFV | | DAT-AC | | PWMH-A | |
| --- | --- | --- | --- | --- | --- | --- |
|  | B | P | B | P | B | P |
| Digit span backward | -0.07 | 0.306 | 0.09 | 0.176 | -0.18 | **0.008** |
| K-BNT | 0.20 | **0.002** | 0.12 | 0.066 | -0.13 | 0.042 |
| RCFT copy | 0.12 | 0.054 | 0.20 | **0.001** | -0.06 | 0.343 |
| SVLT immediate recall | 0.22 | **<0.001** | 0.17 | **0.005** | -0.08 | 0.194 |
| SVLT delayed recall | 0.23 | **<0.001** | 0.08 | 0.177 | -0.14 | 0.037 |
| SVLT recognition | 0.17 | **0.008** | 0.06 | 0.343 | -0.07 | 0.292 |
| RCFT immediate recall | 0.24 | **<0.001** | 0.10 | 0.079 | -0.08 | 0.168 |
| RCFT delayed recall | 0.22 | **<0.001** | 0.11 | 0.055 | -0.07 | 0.232 |
| RCFT recognition | 0.22 | **<0.001** | 0.11 | 0.087 | -0.10 | 0.124 |
| COWAT animal | 0.28 | **<0.001** | 0.10 | 0.094 | -0.20 | **0.002** |
| COWAT phonemic | 0.12 | 0.065 | 0.11 | 0.105 | -0.09 | 0.199 |
| Stroop color reading | 0.23 | **<0.001** | 0.17 | **0.006** | -0.17 | **0.010** |

Multivariable general linear models were used to investigate the effects of BFV, DAT-AC, and PWMH-A on cognition, after controlling for age, sex, education, hypertension, diabetes mellitus, dyslipidemia, number of microbleeds, number of lacunae, and intracranial volume. Significant *P*-values are shown in boldface after false discovery rate correction for multiple comparisons of the regression analyses of 12 tests. Abbreviations: B, standardized β coefficient; BFV, basal forebrain volume; COWAT, Controlled Oral Word Association Test; DAT-AC, dopamine transporter uptake in the anterior caudate; K-BNT, Korean version of the Boston Naming Test; PWMH-A, anterior periventricular white matter hyperintensities; RCFT, Rey–Osterrieth Complex Figure Test; SVLT, Seoul Verbal Learning Test

**Supplementary Table 12.** Effect of BF volume and WMHs on striatal DAT uptake in the non-AD/non-DLB, AD, DLB, and AD/DLB subgroups

|  | BF volume | | PWMH-A | | PWMH-P | | DWMH-A | | DWMH-P | |
| --- | --- | --- | --- | --- | --- | --- | --- | --- | --- | --- |
|  | B | P | B | P | B | P | B | P | B | P |
| **Non-AD/non-DLB** |  |  |  |  |  |  |  |  |  |  |
| DAT-AP | 0.13 | 0.262 | 0.13 | 0.393 | 0.23 | 0.128 | 0.09 | 0.595 | 0.18 | 0.159 |
| DAT-PP | 0.08 | 0.524 | 0.00 | 0.995 | 0.13 | 0.409 | 0.09 | 0.621 | 0.11 | 0.417 |
| DAT-AC | 0.14 | 0.217 | -0.09 | 0.550 | 0.15 | 0.329 | 0.03 | 0.867 | 0.15 | 0.218 |
| DAT-PC | 0.11 | 0.352 | -0.12 | 0.439 | 0.15 | 0.339 | 0.04 | 0.831 | 0.14 | 0.268 |
| **AD** |  |  |  |  |  |  |  |  |  |  |
| DAT-AP | 0.15 | 0.206 | -0.09 | 0.547 | -0.25 | 0.099 | -0.15 | 0.251 | -0.19 | 0.141 |
| DAT-PP | 0.23 | 0.053 | -0.07 | 0.635 | -0.15 | 0.312 | -0.12 | 0.337 | -0.14 | 0.277 |
| DAT-AC | 0.26 | 0.020 | -0.11 | 0.411 | -0.21 | 0.143 | -0.15 | 0.211 | -0.15 | 0.221 |
| DAT-PC | 0.28 | 0.019 | 0.03 | 0.827 | -0.05 | 0.730 | -0.02 | 0.865 | -0.02 | 0.844 |
| **DLB** |  |  |  |  |  |  |  |  |  |  |
| DAT-AP | 0.28 | **0.004** | -0.14 | 0.167 | -0.01 | 0.954 | -0.15 | 0.122 | -0.07 | 0.442 |
| DAT-PP | 0.28 | **0.005** | -0.13 | 0.207 | 0.02 | 0.875 | -0.09 | 0.356 | -0.03 | 0.735 |
| DAT-AC | 0.31 | **0.001** | -0.20 | 0.035 | -0.05 | 0.609 | -0.20 | 0.024 | -0.13 | 0.124 |
| DAT-PC | 0.29 | **0.002** | -0.16 | 0.080 | -0.01 | 0.898 | -0.18 | 0.045 | -0.11 | 0.187 |
| **AD/DLB** |  |  |  |  |  |  |  |  |  |  |
| DAT-AP | 0.04 | 0.702 | -0.10 | 0.357 | -0.11 | 0.288 | -0.19 | 0.065 | -0.05 | 0.582 |
| DAT-PP | -0.13 | 0.195 | -0.01 | 0.957 | 0.00 | 0.996 | -0.10 | 0.339 | 0.01 | 0.907 |
| DAT-AC | 0.13 | 0.163 | -0.25 | 0.014 | -0.23 | 0.020 | -0.20 | 0.040 | -0.16 | 0.103 |
| DAT-PC | 0.15 | 0.128 | -0.24 | 0.022 | -0.16 | 0.120 | -0.18 | 0.070 | -0.08 | 0.397 |
| **DLB+AD/DLB** |  |  |  |  |  |  |  |  |  |  |
| DAT-AP | 0.17 | **0.010** | -0.10 | 0.139 | -0.04 | 0.594 | -0.14 | 0.033 | -0.05 | 0.406 |
| DAT-PP | 0.09 | 0.213 | -0.07 | 0.301 | 0.01 | 0.845 | -0.09 | 0.173 | -0.01 | 0.889 |
| DAT-AC | 0.25 | **<0.001** | -0.20 | **0.003** | -0.10 | 0.114 | -0.17 | **0.008** | -0.12 | 0.059 |
| DAT-PC | 0.23 | **<0.001** | -0.17 | 0.011 | -0.05 | 0.475 | -0.14 | 0.026 | -0.07 | 0.271 |

Univariable general linear models were used to investigate the effects of BF volume and WMHs on striatal DAT uptake after controlling for age, sex, education, HTN, DM, dyslipidemia, number of microbleeds, number of lacunes, and intracranial volume. Significant P-values are shown in boldface after false discovery rate correction for multiple comparisons of regressions analyses for 20 tests in each subgroup. Abbreviations: AC, anterior caudate; AP, anterior putamen; BF, basal forebrain; DAT, dopamine transporter uptake; DWMH-A, anterior deep white matter hyperintensities; DWMH-P, posterior DWMH; PC, posterior caudate; PP, posterior putamen; PWMH-A, anterior periventricular WMH; PWMH-P, posterior PWMH.

**Supplementary Table 13**. Effect of BF volume, DAT-AC, and vascular factors on WMHs in the non-AD/non-DLB, AD, DLB, and AD/DLB subgroups and the combined DLB+AD/DLB subgroup

|  | PWMH-A | | PWMH-P | | DWMH-A | | DWMH-P | |
| --- | --- | --- | --- | --- | --- | --- | --- | --- |
|  | B | *P* | B | *P* | B | *P* | B | *P* |
| **Non-AD/non-DLB** |  |  |  |  |  |  |  |  |
| BF volume | -0.10 | 0.329 | 0.04 | 0.697 | -0.03 | 0.727 | 0.05 | 0.641 |
| DAT-AC | -0.04 | 0.700 | 0.11 | 0.340 | 0.03 | 0.741 | 0.15 | 0.265 |
| Hypertension | 0.11 | 0.278 | 0.02 | 0.806 | 0.08 | 0.377 | 0.00 | 0.995 |
| Diabetes mellitus | 0.00 | 0.993 | 0.05 | 0.598 | -0.03 | 0.740 | 0.06 | 0.577 |
| Dyslipidemia | 0.19 | 0.069 | 0.19 | 0.061 | 0.09 | 0.357 | 0.10 | 0.387 |
| Microbleeds | 0.23 | 0.019 | 0.20 | 0.034 | 0.16 | 0.065 | 0.03 | 0.793 |
| Lacunae | 0.52 | **<0.001** | 0.62 | **<0.001** | 0.74 | **<0.001** | 0.63 | **<0.001** |
| **AD** |  |  |  |  |  |  |  |  |
| BF volume | -0.08 | 0.456 | -0.01 | 0.947 | -0.05 | 0.678 | 0.06 | 0.658 |
| DAT-AC | -0.07 | 0.565 | -0.16 | 0.168 | -0.14 | 0.281 | -0.17 | 0.197 |
| Hypertension | 0.16 | 0.162 | 0.17 | 0.112 | 0.15 | 0.232 | 0.06 | 0.663 |
| Diabetes mellitus | 0.06 | 0.590 | -0.07 | 0.499 | -0.03 | 0.790 | -0.02 | 0.835 |
| Dyslipidemia | -0.09 | 0.422 | 0.00 | 0.980 | 0.00 | 0.969 | 0.05 | 0.710 |
| Microbleeds | 0.32 | **0.003** | 0.38 | **<0.001** | 0.34 | **0.005** | 0.33 | **0.006** |
| Lacunae | 0.26 | **0.017** | 0.24 | **0.020** | 0.21 | 0.082 | 0.07 | 0.557 |
| **DLB** |  |  |  |  |  |  |  |  |
| BF volume | -0.03 | 0.754 | -0.09 | 0.354 | 0.00 | 0.983 | -0.07 | 0.458 |
| DAT-AC | -0.16 | 0.054 | -0.02 | 0.826 | -0.19 | 0.031 | -0.12 | 0.209 |
| Hypertension | 0.05 | 0.475 | 0.12 | 0.119 | 0.12 | 0.144 | 0.14 | 0.102 |
| Diabetes mellitus | 0.07 | 0.358 | -0.01 | 0.877 | -0.03 | 0.677 | -0.11 | 0.187 |
| Dyslipidemia | -0.04 | 0.597 | -0.09 | 0.278 | -0.09 | 0.247 | -0.12 | 0.156 |
| Microbleeds | 0.21 | **0.007** | 0.23 | **0.004** | 0.40 | **<0.001** | 0.23 | **0.005** |
| Lacunae | 0.27 | **0.001** | 0.19 | 0.029 | 0.05 | 0.546 | 0.15 | 0.094 |
| **AD/DLB** |  |  |  |  |  |  |  |  |
| BF volume | -0.14 | 0.112 | -0.05 | 0.582 | 0.03 | 0.772 | 0.04 | 0.676 |
| DAT-AC | -0.21 | 0.025 | -0.22 | 0.026 | -0.20 | 0.039 | -0.17 | 0.096 |
| Hypertension | 0.09 | 0.291 | 0.01 | 0.886 | 0.01 | 0.899 | 0.04 | 0.671 |
| Diabetes mellitus | 0.03 | 0.724 | 0.09 | 0.325 | -0.06 | 0.491 | 0.05 | 0.576 |
| Dyslipidemia | 0.15 | 0.077 | 0.10 | 0.256 | 0.20 | 0.026 | 0.07 | 0.461 |
| Microbleeds | 0.32 | **<0.001** | 0.36 | **<0.001** | 0.38 | **<0.001** | 0.20 | **0.022** |
| Lacunae | 0.28 | **0.001** | 0.25 | **0.005** | 0.28 | **0.002** | 0.35 | **<0.001** |
| **DLB+AD/DLB** |  |  |  |  |  |  |  |  |
| BF volume | -0.07 | 0.242 | -0.06 | 0.339 | 0.02 | 0.784 | 0.00 | 0.951 |
| DAT-AC | -0.16 | **0.008** | -0.08 | 0.195 | -0.18 | **0.008** | -0.12 | 0.070 |
| Hypertension | 0.08 | 0.147 | 0.09 | 0.106 | 0.12 | 0.050 | 0.12 | 0.053 |
| Diabetes mellitus | 0.02 | 0.661 | 0.02 | 0.749 | -0.06 | 0.337 | -0.04 | 0.537 |
| Dyslipidemia | 0.03 | 0.596 | -0.01 | 0.847 | 0.02 | 0.686 | -0.03 | 0.673 |
| Microbleeds | 0.24 | **<0.001** | 0.28 | **<0.001** | 0.30 | **<0.001** | 0.19 | **0.002** |
| Lacunae | 0.31 | **<0.001** | 0.23 | **<0.001** | 0.20 | **0.001** | 0.25 | **<0.001** |

General linear models were used to investigate the effects of BF, striatal DAT, and vascular factors on WMHs after controlling for age, sex, level of education, and intracranial volume. Significant *P*-values are shown in boldface after false discovery rate correction of the four regression analyses for multiple comparisons across the four outcomes. Abbreviations: B, standardized β coefficient; AC, anterior caudate; AP, anterior putamen; BF, basal forebrain; DAT, dopamine transporter; DWMH-A, anterior deep white matter hyperintensities; DWMH-P, posterior DWMH; PC, posterior caudate; PP, posterior putamen; PWMH-A, anterior periventricular white matter hyperintensities; PWMH-P, posterior periventricular white matter hyperintensities.

**Supplementary Table 14**. Effect of BF volume, DAT-AC, and anterior PWMHs on DLB features in the non-AD/non-DLB, AD, DLB, and AD/DLB subgroups and the combined DLB+AD/DLB subgroup

|  | BF volume |  | DAT-AC |  | PWMH-A |  |
| --- | --- | --- | --- | --- | --- | --- |
|  | OR (95% CI) | *P* | OR (95% CI) | *P* | OR (95% CI) | *P* |
| **DLB** |  |  |  |  |  |  |
| Cognitive fluctuation | 0.98 (0.96~1.00) | **0.041** | 0.95 (0.69~1.32) | 0.775 | 1.00 (1.00~1.00) | 0.263 |
| Visual hallucination | 0.98 (0.95~1.00) | **0.043** | 0.74 (0.53~1.01) | 0.066 | 1.00 (1.00~1.00) | 0.951 |
| RBD | 1.00 (0.98~1.02) | 0.875 | 0.68 (0.49~0.91) | **0.011** | 0.99 (0.99~1.00) | **0.035** |
| **AD/DLB** |  |  |  |  |  |  |
| Cognitive fluctuation | 0.96 (0.92~0.99) | **0.016** | 0.69 (0.32~1.36) | 0.306 | 1.00 (1.00~1.00) | 0.399 |
| Visual hallucination | 0.98 (0.94~1.01) | 0.173 | 0.59 (0.34~0.99) | 0.051 | 1.00 (1.00~1.00) | 0.102 |
| RBD | 1.02 (1.00~1.04) | 0.128 | 1.21 (0.82~1.81) | 0.340 | 1.00 (1.00~1.00) | 0.536 |
| **DLB+AD/DLB** |  |  |  |  |  |  |
| Cognitive fluctuation | 0.97 (0.96~0.99) | **0.001** | 0.89 (0.68~1.17) | 0.412 | 1.00 (1.00~1.00) | 0.773 |
| Visual hallucination | 0.98 (0.97~1.00) | 0.058 | 0.68 (0.51~0.88) | **0.004** | 1.00 (1.00~1.00) | 0.212 |
| RBD | 1.01 (0.99~1.02) | 0.238 | 0.83 (0.67~1.03) | 0.101 | 1.00 (1.00~1.00) | 0.323 |

Data are the results of logistic regression models of DLB clinical features (cognitive fluctuation, visual hallucination, and RBD) using BF volume, DAT-AC, and PWMH-A as predictors after controlling for age, sex, level of education, HTN, DM, dyslipidemia, number of microbleeds, number of lacunae, and intracranial volume. Abbreviations: BF, basal forebrain; CI, confidence interval; DAT-AC, dopamine transporter uptake in the anterior caudate; DLB, dementia with Lewy bodies; OR, odds ratio; PWMH-A, anterior periventricular white matter hyperintensities; RBD, rapid eye movement sleep behavior disorder.

**Supplementary Table 15**. Effect of BF volume, DAT-AC, and anterior PWMH on parkinsonism in the non-AD/non-DLB, AD, DLB, and AD/DLB subgroups and the combined DLB+AD/DLB subgroup

|  | BF volume | | DAT-AC | | PWMH-A | |
| --- | --- | --- | --- | --- | --- | --- |
|  | B | *P* | B | *P* | B | *P* |
| **Non-AD/non-DLB** |  |  |  |  |  |  |
| Total UPDRS motor score | 0.10 | 0.431 | -0.27 | 0.068 | -0.35 | **0.041** |
| Axial | 0.17 | 0.190 | 0.02 | 0.909 | -0.15 | 0.348 |
| Bradykinesia | 0.13 | 0.309 | -0.41 | **0.007** | -0.33 | 0.054 |
| Rigidity | -0.08 | 0.565 | -0.16 | 0.285 | -0.45 | **0.011** |
| Tremor | -0.02 | 0.877 | -0.11 | 0.504 | -0.18 | 0.325 |
| **AD** |  |  |  |  |  |  |
| Total UPDRS motor score | -0.18 | 0.217 | 0.13 | 0.401 | 0.07 | 0.664 |
| Axial | -0.15 | 0.270 | 0.07 | 0.621 | 0.21 | 0.191 |
| Bradykinesia | -0.09 | 0.519 | 0.06 | 0.704 | 0.14 | 0.420 |
| Rigidity | -0.06 | 0.664 | 0.08 | 0.608 | 0.11 | 0.502 |
| Tremor | 0.11 | 0.464 | 0.11 | 0.464 | -0.18 | 0.290 |
| **DLB** |  |  |  |  |  |  |
| Total UPDRS motor score | -0.09 | 0.376 | -0.29 | **0.005** | -0.11 | 0.300 |
| Axial | -0.01 | 0.924 | -0.14 | 0.170 | 0.02 | 0.823 |
| Bradykinesia | -0.14 | 0.185 | -0.27 | **0.008** | -0.16 | 0.136 |
| Rigidity | -0.01 | 0.914 | -0.34 | **0.001** | -0.09 | 0.396 |
| Tremor | -0.14 | 0.173 | -0.12 | 0.225 | 0.08 | 0.454 |
| **AD/DLB** |  |  |  |  |  |  |
| Total UPDRS motor score | -0.12 | 0.253 | -0.13 | 0.211 | 0.28 | **0.016** |
| Axial | -0.15 | 0.148 | -0.06 | 0.555 | 0.16 | 0.166 |
| Bradykinesia | -0.12 | 0.259 | -0.14 | 0.184 | 0.32 | **0.005** |
| Rigidity | -0.01 | 0.960 | -0.14 | 0.201 | 0.22 | 0.070 |
| Tremor | -0.04 | 0.750 | 0.00 | 0.972 | 0.08 | 0.525 |
| **DLB+AD/DLB** |  |  |  |  |  |  |
| Total UPDRS motor score | -0.10 | 0.178 | -0.22 | **0.003** | 0.05 | 0.496 |
| Axial | -0.05 | 0.491 | -0.08 | 0.257 | 0.10 | 0.181 |
| Bradykinesia | -0.14 | 0.063 | -0.21 | **0.005** | 0.04 | 0.605 |
| Rigidity | -0.01 | 0.893 | -0.26 | **< 0.001** | 0.03 | 0.685 |
| Tremor | -0.08 | 0.306 | -0.07 | 0.342 | 0.05 | 0.497 |

General linear models were used to investigate the effects of BF volume, DAT-AC, and PWMH-A on parkinsonism after controlling for age, sex, education, hypertension, diabetes mellitus, dyslipidemia, number of microbleeds, number of lacunae, and intracranial volume. Significant *P*-values are shown in boldface after false discovery rate correction for multiple comparisons of regression analyses for four subscores of the UPDRS motor score. Abbreviations: B, standardized β coefficient; BF, basal forebrain; DAT-AC, dopamine transporter uptake in the anterior caudate; PWMH-A, anterior periventricular white matter hyperintensities; UPDRS, Unified Parkinson’s Disease Rating Scale.

**Supplementary Table 16.** Multivariable analysis of the effect of BF volume, DAT-AC, and PWMH-A on cognition in the non-AD/non-DLB, AD, DLB, and AD/DLB subgroups and the combined DLB+AD/DLB subgroup

|  | BF volume | | DAT-AC | | PWMH-A | |
| --- | --- | --- | --- | --- | --- | --- |
|  | B | P | B | P | B | P |
| **Non-AD/non-DLB** |  |  |  |  |  |  |
| Digit span backward | -0.15 | 0.317 | 0.10 | 0.529 | 0.04 | 0.832 |
| K-BNT | 0.03 | 0.860 | 0.19 | 0.247 | -0.03 | 0.887 |
| RCFT copy | 0.13 | 0.334 | 0.21 | 0.143 | 0.01 | 0.973 |
| SVLT immediate recall | 0.09 | 0.503 | 0.41 | **0.006** | -0.04 | 0.785 |
| SVLT delayed recall | 0.07 | 0.602 | 0.43 | **0.005** | -0.12 | 0.478 |
| SVLT recognition | 0.24 | 0.094 | 0.24 | 0.128 | 0.03 | 0.856 |
| RCFT immediate recall | 0.02 | 0.877 | 0.33 | 0.036 | 0.11 | 0.527 |
| RCFT delayed recall | -0.07 | 0.591 | 0.39 | 0.014 | 0.01 | 0.939 |
| RCFT recognition | -0.07 | 0.639 | 0.25 | 0.145 | -0.08 | 0.674 |
| COWAT semantic | 0.02 | 0.874 | 0.38 | 0.020 | 0.00 | 0.995 |
| COWAT phonemic | 0.00 | 0.985 | 0.20 | 0.219 | 0.01 | 0.948 |
| Stroop color reading | 0.14 | 0.304 | 0.10 | 0.504 | -0.12 | 0.537 |
| **AD** |  |  |  |  |  |  |
| Digit span backward | -0.23 | 0.110 | -0.01 | 0.929 | -0.13 | 0.423 |
| K-BNT | 0.08 | 0.540 | 0.11 | 0.454 | -0.40 | 0.008 |
| RCFT copy | 0.11 | 0.456 | -0.07 | 0.639 | -0.05 | 0.777 |
| SVLT immediate recall | 0.06 | 0.635 | 0.10 | 0.462 | 0.01 | 0.943 |
| SVLT delayed recall | 0.11 | 0.390 | 0.11 | 0.425 | -0.04 | 0.772 |
| SVLT recognition | -0.02 | 0.846 | 0.08 | 0.526 | -0.02 | 0.872 |
| RCFT immediate recall | 0.11 | 0.351 | 0.03 | 0.798 | 0.06 | 0.659 |
| RCFT delayed recall | 0.08 | 0.498 | 0.00 | 0.981 | -0.03 | 0.823 |
| RCFT recognition | 0.28 | 0.030 | -0.11 | 0.397 | -0.04 | 0.753 |
| COWAT semantic | 0.35 | 0.006 | 0.20 | 0.137 | -0.02 | 0.871 |
| COWAT phonemic | -0.06 | 0.668 | 0.19 | 0.208 | 0.12 | 0.451 |
| Stroop color reading | -0.03 | 0.795 | 0.00 | 0.975 | -0.07 | 0.628 |
| **DLB** |  |  |  |  |  |  |
| Digit span backward | -0.09 | 0.372 | 0.03 | 0.740 | -0.24 | 0.013 |
| K-BNT | 0.16 | 0.110 | 0.01 | 0.939 | -0.12 | 0.222 |
| RCFT copy | 0.08 | 0.432 | 0.19 | 0.045 | 0.01 | 0.908 |
| SVLT immediate recall | 0.29 | **0.004** | 0.08 | 0.377 | -0.05 | 0.631 |
| SVLT delayed recall | 0.25 | 0.017 | 0.02 | 0.809 | -0.13 | 0.214 |
| SVLT recognition | 0.14 | 0.207 | -0.02 | 0.882 | -0.04 | 0.679 |
| RCFT immediate recall | 0.29 | **0.004** | 0.06 | 0.511 | -0.10 | 0.296 |
| RCFT delayed recall | 0.21 | 0.041 | 0.11 | 0.261 | -0.06 | 0.545 |
| RCFT recognition | 0.12 | 0.226 | 0.12 | 0.208 | -0.11 | 0.279 |
| COWAT semantic | 0.19 | 0.061 | 0.07 | 0.444 | -0.22 | 0.027 |
| COWAT phonemic | 0.17 | 0.107 | 0.01 | 0.947 | -0.10 | 0.343 |
| Stroop color reading | 0.18 | 0.082 | 0.18 | 0.074 | -0.19 | 0.061 |
| **AD/DLB** |  |  |  |  |  |  |
| Digit span backward | 0.00 | 0.967 | 0.03 | 0.792 | -0.20 | 0.083 |
| K-BNT | 0.38 | **<0.001** | 0.20 | 0.049 | -0.01 | 0.916 |
| RCFT copy | 0.21 | 0.031 | 0.22 | 0.029 | -0.19 | 0.068 |
| SVLT immediate recall | 0.15 | 0.122 | 0.13 | 0.189 | -0.31 | **0.005** |
| SVLT delayed recall | 0.19 | 0.065 | 0.12 | 0.265 | -0.29 | **0.009** |
| SVLT recognition | 0.21 | 0.047 | 0.06 | 0.589 | -0.18 | 0.108 |
| RCFT immediate recall | 0.23 | **0.015** | 0.14 | 0.164 | -0.20 | 0.058 |
| RCFT delayed recall | 0.29 | **0.002** | 0.18 | 0.062 | -0.15 | 0.129 |
| RCFT recognition | 0.29 | **0.006** | 0.17 | 0.112 | -0.21 | 0.058 |
| COWAT semantic | 0.34 | **0.001** | -0.01 | 0.935 | -0.30 | **0.006** |
| COWAT phonemic | 0.21 | 0.053 | 0.08 | 0.491 | -0.22 | 0.068 |
| Stroop color reading | 0.40 | **<0.001** | 0.08 | 0.469 | -0.20 | 0.072 |
| **DLB+AD/DLB** |  |  |  |  |  |  |
| Digit span backward | -0.03 | 0.658 | 0.05 | 0.489 | -0.21 | **0.005** |
| K-BNT | 0.23 | **0.001** | 0.08 | 0.238 | -0.08 | 0.290 |
| RCFT copy | 0.13 | 0.056 | 0.21 | **0.002** | -0.07 | 0.331 |
| SVLT immediate recall | 0.26 | **<0.001** | 0.11 | 0.102 | -0.13 | 0.059 |
| SVLT delayed recall | 0.26 | **<0.001** | 0.05 | 0.494 | -0.18 | **0.013** |
| SVLT recognition | 0.21 | **0.003** | 0.01 | 0.866 | -0.10 | 0.175 |
| RCFT immediate recall | 0.28 | **<0.001** | 0.09 | 0.165 | -0.13 | 0.054 |
| RCFT delayed recall | 0.26 | **<0.001** | 0.13 | 0.058 | -0.10 | 0.161 |
| RCFT recognition | 0.20 | **0.005** | 0.14 | 0.050 | -0.14 | 0.061 |
| COWAT semantic | 0.26 | **<0.001** | 0.04 | 0.512 | -0.25 | **0.001** |
| COWAT phonemic | 0.16 | **0.027** | 0.05 | 0.519 | -0.16 | 0.039 |
| Stroop color reading | 0.28 | **<0.001** | 0.14 | 0.050 | -0.19 | **0.007** |

Multivariable general linear models were used to investigate the effects of BF volume, DAT-AC, and PWMH-A on cognition, after controlling for age, sex, education, hypertension, diabetes mellitus, dyslipidemia, number of microbleeds, number of lacunae, and intracranial volume. Significant *P*-values are shown in boldface after false discovery rate correction for multiple comparisons of the regression analyses of 12 tests. Abbreviations: B, standardized β coefficient; BF, basal forebrain; COWAT, Controlled Oral Word Association Test; DAT-AC, dopamine transporter uptake in the anterior caudate; K-BNT, Korean version of the Boston Naming Test; PWMH-A, anterior periventricular white matter hyperintensities; RCFT, Rey–Osterrieth Complex Figure Test; SVLT, Seoul Verbal Learning Test.

**Supplementary Table 17.** Independent effect of BFV, striatal DAT uptake, and vascular factors on WMHs after adjusting for presences of AD and DLB

|  | PWMH-A | | PWMH-P | | DWMH-A | | DWMH-P | |
| --- | --- | --- | --- | --- | --- | --- | --- | --- |
| Predictors | B | P | B | P | B | P | B | P |
| **Multivariable analysis** | |  |  |  |  |  |  |  |
| AD | 0.002 | 0.957 | 0.04 | 0.359 | 0.07 | 0.160 | 0.08 | 0.109 |
| DLB | 0.01 | 0.766 | -0.01 | 0.851 | -0.05 | 0.296 | -0.08 | 0.110 |
| BFV | -0.08 | 0.086 | -0.03 | 0.533 | -0.01 | 0.867 | 0.04 | 0.452 |
| DAT-AC | -0.13 | **0.008** | -0.07 | 0.157 | -0.12 | **0.026** | -0.09 | 0.129 |
| Hypertension | 0.10 | **0.021** | 0.10 | **0.028** | 0.11 | **0.027** | 0.08 | 0.107 |
| Diabetes mellitus | 0.03 | 0.439 | 0.02 | 0.717 | -0.04 | 0.381 | -0.02 | 0.760 |
| Dyslipidemia | 0.03 | 0.469 | 0.02 | 0.578 | 0.04 | 0.458 | 0.04 | 0.469 |
| Microbleeds | 0.27 | **< 0.001** | 0.29 | **< 0.001** | 0.28 | **< 0.001** | 0.19 | **< 0.001** |
| Lacunes | 0.33 | **< 0.001** | 0.29 | **< 0.001** | 0.25 | **< 0.001** | 0.26 | **< 0.001** |

General linear models were used to investigate independent effects BFV, DAT-AC and vascular factors on WMHs after controlling for the presences of AD and DLB, age, sex, levels of education and intracranial volume. Abbreviations: AD, Alzheimer’s disease; B, standardized beta coefficient; BFV, basal forebrain volume; DAT-AC; dopamine transporter uptake in the anterior caudate; DLB, dementia with Lewy bodies; hyperintensities; DWMH-A, anterior deep white matter hyperintensities; DWMH-P, posterior DWMH; PWMH-A, anterior periventricular WMH; PWMH-P, posterior PWMH
